# Dysregulated inflammasome activity in intestinal inflammation – Insights from patients with very early onset IBD

**DOI:** 10.3389/fimmu.2022.1027289

**Published:** 2022-11-29

**Authors:** David Illig, Daniel Kotlarz

**Affiliations:** ^1^ Department of Pediatrics, Dr. von Hauner Children’s Hospital, University Hospital, Ludwig-Maximilians-University (LMU), Munich, Germany; ^2^ Institute of Translational Genomics, Helmholtz Zentrum München, German Research Center for Environmental Health, Neuherberg, Germany

**Keywords:** VEO-IBD, inflammasome, immunodeficiency, pediatrics, genetics, inflammation

## Abstract

Inflammatory bowel disease (IBD) is a multifactorial disorder triggered by imbalances of the microbiome and immune dysregulations in genetically susceptible individuals. Several mouse and human studies have demonstrated that multimeric inflammasomes are critical regulators of host defense and gut homeostasis by modulating immune responses to pathogen- or damage-associated molecular patterns. In the context of IBD, excessive production of pro-inflammatory Interleukin-1β has been detected in patient-derived intestinal tissues and correlated with the disease severity or failure to respond to anti-tumor necrosis factor therapy. Correspondingly, genome-wide association studies have suggested that single nucleotide polymorphisms in inflammasome components might be associated with risk of IBD development. The relevance of inflammasomes in controlling human intestinal homeostasis has been further exemplified by the discovery of very early onset IBD (VEO-IBD) patients with monogenic defects affecting different molecules in the complex regulatory network of inflammasome activity. This review provides an overview of known causative monogenic entities of VEO-IBD associated with altered inflammasome activity. A better understanding of the molecular mechanisms controlling inflammasomes in monogenic VEO-IBD may open novel therapeutic avenues for rare and common inflammatory diseases.

## Inflammasomes – Central coordinators of innate immunity

**Figure 1:**
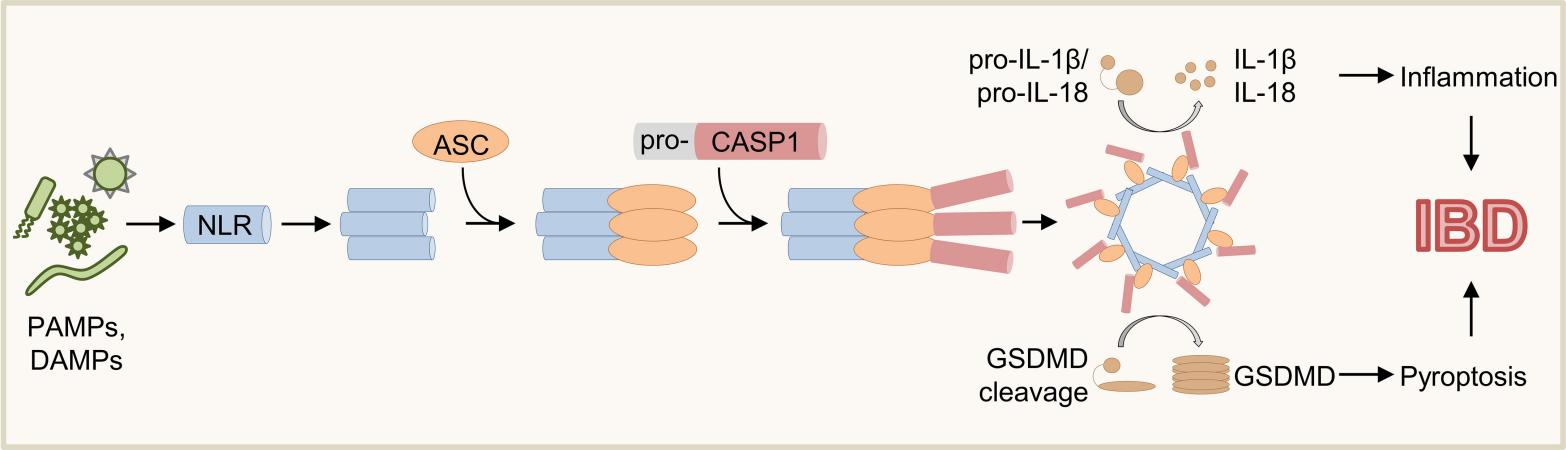
Schematic overview of inflammasome activation. Various PAMPs and DAMPs induce activation of sensor proteins resulting in oligomerization and recruitment of ASC and pro-CASP1. Upon autoproteolysis of pro-CASP1, mature CASP1 cleaves the inflammasome effector molecules pro-IL-1β, pro-IL-18, and GSDMD, which induce inflammation and pyroptosis.

Inflammasomes are multimeric cytosolic protein complexes controlling immune tolerance, inflammation, host defense, cell clearance, and tissue repair (1, 2). The modal composition of inflammasomes based on common adaptors and effectors paired with cell-type specific sensors allows mounting of context-dependent responses to distinct threats (3, 4). As a first step of inflammasome activation, sensor proteins (e.g., Absent in melanoma 2 (AIM2), Nucleotide-binding oligomerization domain, Leucine rich Repeat (NLR) and Pyrin domain (PYD) containing protein (NLRP) 3, NLR family, apoptosis inhibitory protein (NAIP)/NLR family caspase activation and recruitment domain (CARD) domain-containing protein 4 (NLRC4), PYRIN) detect various danger signals including pathogen-associated molecular patterns (PAMPs) or damage-associated molecular patterns (DAMPs) (**Figure 1**) (1, 3, 4). Whereas some sensors are specific to distinct signals (e.g., AIM2, NLRC4), others (e.g., NLRP3) are promiscuous and can respond to a variety of stimuli (1, 4–15). Sensor proteins contain CARD or pyrin domains (PYD) mediating the interaction with adaptors and/or effectors (4, 16). Upon activation, some sensor proteins can directly recruit the effector Caspase (CASP) 1 *via* their CARD (**Figure 1**) (13, 16–19). In contrast, sensor proteins lacking a CARD recruit the adapter protein apoptosis-associated speck-like protein containing a CARD (ASC) *via* interaction of PYD (20–22). In turn, ASC can interact with pro-CASP1 *via* CARD resulting in oligomerization of inflammasome components and activation of pro-CASP1 by autoproteolysis (**Figure 1**) (3, 4, 13, 21, 23, 24). Finally, mature CASP1 cleaves the inflammasome substrates pro-Interleukin (IL)-1β, pro-IL-18 and Gasdermin D (GSDMD) (**Figure 1**) (1, 3, 4, 23, 25–29). While IL-1β and IL-18 trigger activation and recruitment of other immune cells contributing to inflammation and host defense, insertion of mature GSDMD into cell membranes induces pore formation and pyroptosis (**Figure 1**) (1, 3, 4, 25, 26).

Complex regulatory mechanisms on transcriptional and post-translational level are required to facilitate balanced inflammasome-mediated immune responses. On transcriptional level, nuclear factor κ-B (NF-κB)-mediated signaling has been shown to be critical for transcription of central inflammasome components (e.g., *NLRP3* and *IL1B*) upon Toll-like receptor (TLR)-mediated detection of PAMPs or DAMPs (30, 31). This process is often referred to as priming or signal 1 of NLRP3 inflammasomes (1, 30, 31). The subsequent triggering of sensor proteins was termed activation step or signal 2 and can involve post-translational processes. For example, NLRP3 inflammasome activation requires ATP-mediated deubiquitination of NLRP3 by BRCA1/BRCA2-Containing Complex Subunit 3 (BRRC3) but is inhibited by interferon (IFN)-γ-induced nitrosylation (1, 31–34). Furthermore, various kinases were shown to control activity of NLRC4, Pyrin (see also MEFV below), or ASC by phosphorylation (1, 35–38).

## The role of inflammasomes in intestinal inflammation

### Inflammasomes in intestinal epithelial cells

The intestinal epithelial barrier represents the first line of defense against pathogens and is critical in controlling intestinal immunity. Inflammasomes have been shown to play a central role in the defense strategy of intestinal epithelial cells (IEC), which is reflected by the expression of a diverse repertoire of inflammasome sensor proteins including NLRC4, NLRP3, and NLRP6 (39). In contrast to other epithelial cell types, IEC were shown to produce higher levels of IL-18 but less IL-1β indicating that IL-18 has a distinct role in intestinal homeostasis (39–42). For example, IL-18 has been involved in controlling infections by stimulating IFN-γ production from T and NK cells and supporting T_H_1 responses (31, 43, 44). In addition, the induction of epithelial inflammasomes contributes not only to activation of immune cells *via* IL-18 but supports also viral clearance by inducing direct release of IFNs (16, 45). Furthermore, IEC-related inflammasomes stimulate mucus secretion, pyroptosis, or expulsion of infected epithelial cells (16, 46–48).

### Inflammasomes in immune cells

Inflammasomes are primarily known for their function in innate immune cells (e.g., macrophages, granulocytes) and intestinal myeloid cells are the major source of IL-1β in the gut (31). Inflammasome activity in immune cells of the gut is critical for the detection of a wide variety of pathogens (e.g., bacteria, viruses, parasites) and the induction of appropriate host defense mechanisms (4, 16). Pathogen-induced activation of inflammasomes results in production of the pro-inflammatory cytokines IL-1β and IL-18, which induce a cascade of signaling pathways culminating in recruitment of other immune cells (e.g., neutrophils) (4, 16). Upon IL-1β sensing, immune cells produce various pro-inflammatory molecules (e.g., IL-6 and tumor necrosis factor (TNF)-α) fueling inflammation in the gut (31). In adaptive immune cells, IL-1β was shown to induce T cell survival and proliferation as well as increased immunoglobulin production by B cells (31). Furthermore, IL-1β contributes to polarization of T_H_17 cells that are important mediators of intestinal inflammation (49, 50). Although immune cell-derived IL-1β can induce epithelial repair by stimulating renewal of intestinal stem cells, excess IL-1β might amplify intestinal inflammation by increasing epithelial barrier permeability and production of cytokines and chemokines (51–54). In addition to production of cytokines, inflammasome-dependent activation of pyroptosis in immune cells restrains intracellular replication of pathogens in infected immune cells (4, 16, 55). Moreover, inflammasomes were also shown to contribute to discrimination between pathogenic and commensal microbiota in the gastrointestinal tract (16, 56).

### Inflammasomes in infectious diseases affecting the gastrointestinal tract

Various pattern recognition receptor families including the inflammasome sensor proteins of the NLR protein family have evolved in humans to recognize foreign and/or potentially dangerous material. Several pathogens affecting gastrointestinal health have been shown to trigger activation of inflammasomes. For example, NLRP3 inflammasomes contribute to the clearance of various bacterial (e.g., *Helicobacter pylori*, *Campylobacter jejuni*, *Yersinia enterocolitica*) and viral (e.g., adenovirus, enterovirus) species (16, 57–63). Furthermore, NAIP/NLRC4 can be triggered by components (e.g., Flagellin or type 3 secretion system) from various enteric bacterial species including *Escherichia coli, Salmonella enterica*, and *Listeria monocytogenes* (12, 14–16, 64). Moreover, *Clostridium difficile infections*, a major cause for antibiotic-related diarrhea and pseudomembranous colitis, result in toxin-mediated activation of Pyrin inflammasomes and increased IL-1β-dependent tissue damage (16, 65, 66). Mechanistically, infection-induced activation of inflammasomes contributes to pathogen clearance by cytokine-mediated recruitment and activation of immune cells (e.g, neutrophils) and pyroptosis of infected cells limiting pathogen propagation (16, 41, 46, 47, 56). Furthermore, active inflammasomes can limit further uptake of pathogens and can increase pathogen killing of professional phagocytes (16, 67). In IEC, inflammasome activation can lead to expulsion of infected cells into the gastrointestinal lumen, which may hinder pathogens to overcome the intestinal barrier (16, 41, 46, 47).

### Inflammasomes in IBD pathogenesis

Previous studies have indicated that inflammasomes are implicated in IBD pathogenesis, as mucosal IL-1 production is significantly enhanced during active disease (68). Furthermore, higher IL-1β levels were detected in LPS-stimulated peripheral blood mononuclear cells (PBMCs) from patients with Crohn’s disease (CD) and long-standing ulcerative colitis (UC) (69). In line, expression of IL-18 was also shown to be higher in lamina propria mononuclear cells isolated from patients with CD (70, 71). Moreover, IL-1β signatures have been detected in macrophages/monocytes isolated from inflamed intestinal tissues of IBD patients by single-cell transcriptomics and deep immunoprofiling (72). Correspondingly, Liso et al. have recently demonstrated that failure to respond to anti-TNF therapy was associated with increased IL-1β in sera and colonic biopsy specimens from patients with UC (73). Genetic effects on inflammasome dysregulation in IBD susceptibility were suggested by polymorphisms in genes involved in inflammasome activity (e.g., NLRP3, IL-18) (31, 74–76). Additionally, mutations in the NLRP3 regulator CARD8 were shown to result in increased NLRP3 inflammasome activity and CD (77).

The important role of inflammasomes in controlling homeostasis of the intestinal tract has been further demonstrated by amelioration of experimental colitis through blockade of the inflammasome effector molecules IL-1β and IL-18 in different murine models (50, 73, 78, 79). Based on these studies, IL-1 blockade is considered as potential therapy for IBD and is currently being evaluated in a phase II randomized placebo-controlled double-blinded trial for patients with acute severe colitis (80).

## Monogenic VEO-IBD – A powerful model to define key factors controlling inflammasome activity

IBD is a complex disease triggered by environmental factors, immune dysfunctions, epithelial barrier defects, and imbalances of the microbial flora in genetically susceptible individuals (81). In particular, children with rare very early onset IBD (VEO-IBD) show severe and refractory inflammatory conditions different from forms observed in adults (82). Based on the early age of onset and the aggressive phenotype VEO-IBD patients are considered to have a higher genetic susceptibility. In line, >75 distinct single inherited genetic defects have been identified as molecular cause for VEO-IBD (83, 84). Notably, the majority of reported monogenic entities are underlying primary immunodeficiencies and genetic diagnosis has critical implications for the prognosis and therapy of VEO-IBD patients. For example, hematopoietic stem cell transplantation (HSCT) has been established as curative standard of care for VEO-IBD patients associated with inborn errors of immunity (85).

**Figure 2:**
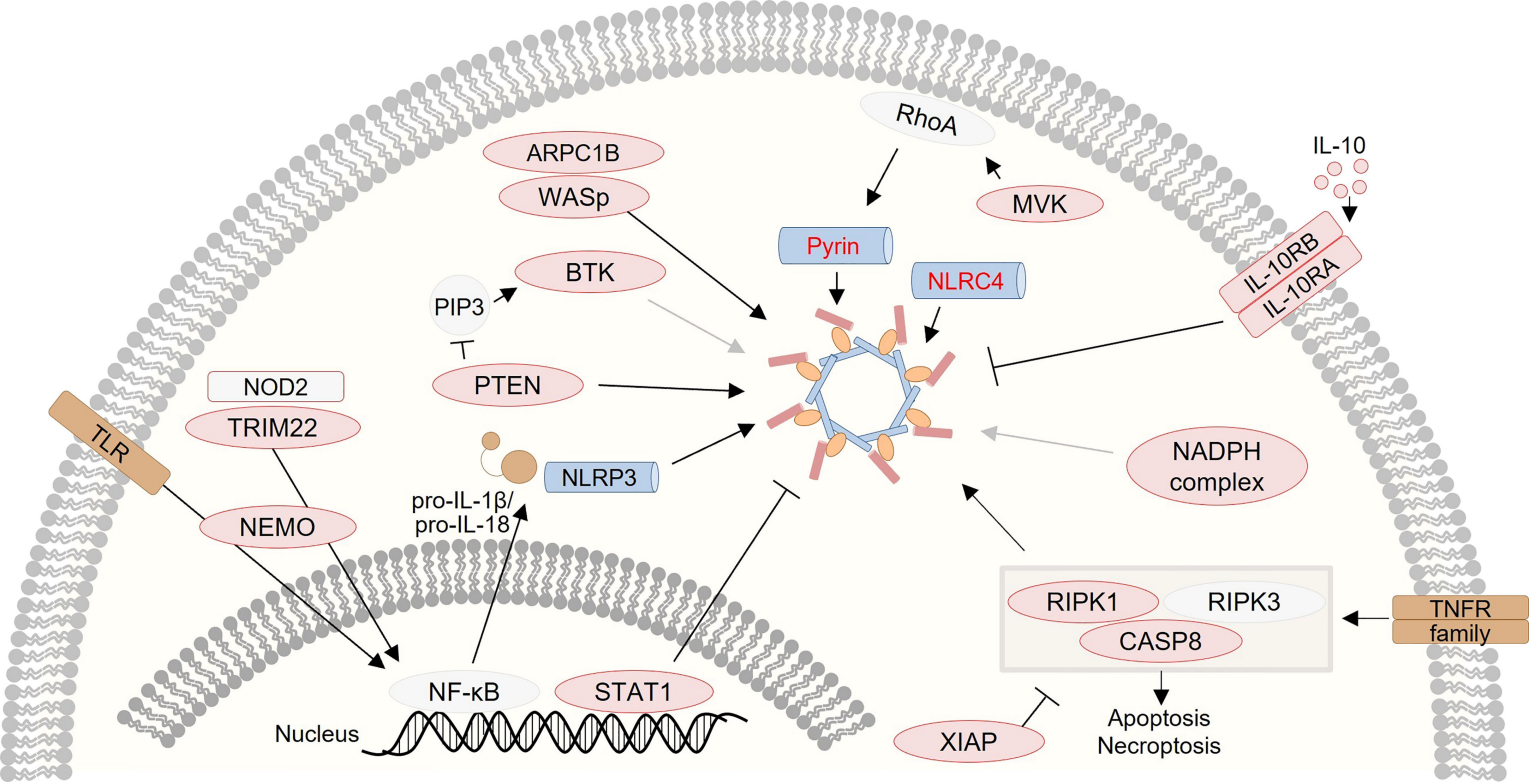
Graphical presentation of a network of inflammasome-associated monogenic defects causing VEO-IBD. Several proteins, that are several candidates for monogenic VEO-IBD (highlighted in red), have been shown to contribute to inflammasome dysregulation *via* various mechanisms.

Notably, several studies on monogenic VEO-IBD have demonstrated that altered inflammasome activity plays a critical role in the pathogenesis of human intestinal inflammation and illustrated that inflammasome plasticity is regulated by complex networks (**Figure 2**). Thus, monogenic VEO-IBD represents a powerful model highlighting critical molecular nodes forming the skeleton of inflammasome regulation. A better understanding of human inflammasome biology will guide the development of personalized therapies for VEO-IBD but will also portray novel concepts for the treatment of common IBD. To stimulate research on inflammasome biology in IBD pathogenesis, we herein aim to provide an overview of genes known to cause (monogenic) IBD and influence inflammasome activity. Therefore, we screened for genetic defects reported in recent position papers (83, 86–89) related to monogenic IBD to summarize links to inflammasome-related genes and -mediated processes (**Table 1**).

**Table 1:** Overview of monogenic forms of IBD associated with inflammasome dysregulation.

Gene	Disease	Effect on IL-1β production	Effect of IL-1 blockade	References
*ADAM17*	Neonatal inflammatory skin and bowel disease	not clear	n.a.	
*BTK*	X-linked agammaglobulinemia 1	context-dependent	+ (mouse)	(90, 91)
*CASP8*	Caspase-8 deficiency	*↑*	n.a.	(92)
*CYBA*	Chronic granulomatous disease	*↑*	+	(78, 93)
*CYBB*	Chronic granulomatous disease	*↑*	+	(78, 93)
*IKBKG*	Anhidrotic X-linked ectodermal dysplasia and immunodeficiency	not clear	n.a.	
*IL10/IL10RA/IL10RB*	IL-10 (receptor) deficiency	*↑*	+	(94–96)
*MEFV*	Familial Mediterranean Fever	*↑*	+	(97–100)
*MVK*	Mevalonate kinase deficiency	*↑*	+	(38, 97, 101, 102)
*NCF1*	Chronic granulomatous disease	*↑*	+	(78, 93)
*NCF2*	Chronic granulomatous disease	*↑*	+	(78, 93)
*NLRC4*	Autoinflammation with infantile enterocolitis	*↑*	IL-18	(103–105)
*PTEN*	PTEN hamartoma tumor syndrome	*↓*	n.a.	(106)
*RIPK1*	RIPK1 deficiency	*↑*	n.a.	(107, 108)
*STAT1*	IPEX-like disease	not clear	n.a.	
*TRIM22*	TRIM22 defect	*↓*	n.a.	(109)
*WAS*	Wiskott-Aldrich syndrome	*↑*	+	(110–112)
*XIAP*	X-linked lymphoproliferative syndrome 2	*↑*	n.a.	(113–115)

Alphabetical list of IBD candidate genes showing effects on IL-1β production as well as the therapeutic efficacy of IL-1 blockade in the corresponding disorder. If a robust phenotype on IL-1β production has been documented the effect is indicated by arrows (up: higher IL-1β production; down: lower IL-1β production). Beneficial effects of IL-1 blockade are indicated by a plus sign. For BTK deficiency, only mouse data are available. For NLRC4 mutations, IL-18 blockade was shown to be effective. N.a., not assessed.

## Inflammasome dysregulation in monogenic VEO-IBD

### Sensor proteins

#### NLRC4

The most direct link between IBD pathogenesis and dysregulated inflammasome activation has been provided by the discovery of patients carrying mutations in genes encoding for sensor proteins. In particular, *de novo* gain-of-function in *NLRC4* could be identified in patients presenting with a range of clinical manifestations of autoinflammation and macrophage activation syndrome, including severe very early onset enterocolitis (103, 104). The reported gain-of-function mutations cause spontaneous oligomerization and activation of the NAIP/NLRC4 inflammasome without the requirement of physiological triggers resulting in spontaneous cleavage of pro-CASP1 and excessive release of IL-1β and IL-18, pyroptosis of macrophages, and chronic inflammation (103, 104). In addition, Steiner et al. have recently identified an autosomal recessive NLRC4 mutation associated with increased IL-1β and IL-18 secretion in a patient with autoinflammation accompanied by diarrhea (116). Although the underlying mechanisms of biallelic NLRC4 deficiency remain elusive, IL-18 blockade was shown to be effective in treatment of NLRC4-mediated macrophage activation syndrome indicating that epithelial-derived IL-18 might be a critical pathomechanistic driver (105). Taken together, patients with germline NLRC4 mutations demonstrate that a tight regulation of NLRC4-associated inflammasomes is necessary to maintain intestinal homeostasis and physiological immune cell function.

#### MEFV

Patients with mutations in the *Mediterranean fever gene* (*MEFV*, encoding for pyrin) have been shown to develop an autoinflammatory syndrome called Familial Mediterranean Fever (FMF) characterized by periodic fever attacks and associated with early-onset IBD-like phenotypes (38, 97, 117, 118). Even though mutations in MEFV are discussed rather as risk factors for VEO-IBD, underlying mechanisms in FMF provide important insights into dysregulated inflammasome activity. In steady state, pyrin molecules are phosphorylated by serine/threonine-protein kinase N (PKN)1/2 allowing binding of the chaperone protein 14-3-3 and maintenance of inactive pyrin (38, 97). In turn, activity of PKN1/2 is controlled by Rho GTPases that are critical regulators of actin cytoskeletons indicating that pyrin-mediated inflammasome activity is coupled to cytoskeleton dynamics (37, 38, 65). Various bacterial toxins (e.g., TcdB from *Clostridium difficile*) cause inhibition of Rho GTPases by post-translational modification, which results in reduced PKN1/2 activity, pyrin phosphorylation, and 14-3-3 recruitment, subsequently leading to increased pyrin activity (38, 65, 97). Similar to other inflammasomes, oligomerized pyrin recruits ASC and CASP1 resulting in inflammation and pyroptosis by inducing cleavage of pro-IL-1β, pro-IL-18 and GSDMD (119). Gain-of-function mutations in *MEFV* can cause increased IL-1β production and levels of IL-1β are indicative of disease activity in FMF patients (97, 98). As first line therapy, FMF can be successfully treated with colchicine by blocking polymerization of microtubuli and maintaining pyrin in an inactive state through subsequent activation of Rho GTPases and PKN1/2 (99, 120–122). Notably, FMF might be also treated using IL-1β antagonists demonstrating the central role of an inflammasome-mediated pathogenesis in FMF (99, 100, 120).

### Inflammasome regulators

#### MVK

Mevalonate kinase (MVK) catalyzes the phosphorylation of mevalonate, which is a critical step in the biosynthesis of cholesterol as well as isopentenyl diphosphate and other polyisoprenoid metabolites (97, 123). Furthermore, the mevalonate pathway also produces precursors of geranylgeranyl pyrophosphate required for prenylation of proteins (101). Notably, prenylation is a critical post-translational modification of small Rho GTPases, which are important for the regulation of the pyrin inflammasome (see also *MEFV*) (38, 65, 97, 101). In patients with MVK deficiency, loss-of-function mutations impair production of mevalonate metabolites resulting in accumulation of metabolic precursors and lack of products like geranylgeranyl pyrophosphate (38, 65, 97, 101). The underlying mechanisms of enhanced inflammasome activity in MVK deficiency are not fully understood, but defective protein prenylation of small GTPases causes reduced pyrin phosphorylation and thereby induces spontaneous activation of pyrin inflammasomes (38, 65, 97, 101). Similar to MEFV, patients with MVK deficiency suffer from an autoinflammatory syndrome characterized by recurrent episodes of fever, arthralgia, lymphadenopathy, and splenomegaly (38, 124, 125). Of note, MVK-deficient patients can also present with very-early onset diarrhea and abdominal pain reminiscent of IBD (38, 124, 125). Interestingly, the disease severity of MVK deficiency is dependent on the residual activity of mutated MVK (38, 124). Severe forms of MVD present as mevalonic aciduria associated with developmental delay and severe systemic inflammation (126). Analogous to FMF patients, MVK-deficient patients with VEO-IBD have been successfully treated by biologics blocking IL-1β signaling leading to improved endoscopic, histologic and laboratory parameters of inflammation (102).

#### IKBKG

NF-κB signaling is a critical cellular signaling pathway in human cells controlling pleiotropic functions such as inflammatory responses, cell stress, cell survival, and cell growth (127–129). Several studies have demonstrated that NF-κB signaling is critical for the expression of NLRP3 inflammasome components (e.g., *NLRP3, IL1B*) in response to various danger signals (priming step) (30, 31). In unstimulated conditions, NF-κB is inhibited by binding to the inhibitor of κB (IκB) (130–132). Upon cellular activation, IκB proteins are phosphorylated by the IκB kinase (IKK) complex (IKKα, IKKβ, IKKγ/NF-κB essential modulator (NEMO)) releasing IκB and enabling NF-κB-mediated signaling (132–136). In males, hypomorphic mutations in *NEMO*, a gene with X-linked inheritance encoding a regulatory subunit of the IKK complex, cause immunodeficiency and hypohidrotic ectodermal dysplasia associated with severe bacterial, viral, and fungal infections (137–139). Many NEMO-deficient patients further present with VEO-IBD characterized by intractable diarrhea and failure-to-thrive (137, 138). On a molecular level, NEMO deficiency causes aberrant TLR-, TNFR-, and IL-1R-mediated signaling impairing critical immune cell functions in response to infection (137). Of note, HSCT was shown to cure immunodeficiency and susceptibility to infections in patients with NEMO deficiency, but failed to cure intestinal inflammation indicating an important role of NEMO and NF-κB signaling in controlling intestinal epithelial cell homeostasis (138). In fact, NEMO was shown to be a critical regulator of TNF-mediated and RIPK1-dependent cell death in intestinal epithelium and NEMO-deficient epithelial cells displayed increased cell death as well as reduced production of antimicrobial molecules leading to increased permeability of the intestinal barrier for luminal microbiota and to intestinal inflammation (140, 141).

Genetic variants disturbing NF-κB signaling are obvious candidates causing inflammasome activation defects. In line, Greten et al. could show that inhibition or deletion of IKKβ results in reduced expression of IL-1β mRNA and immature protein upon LPS stimulation in mouse macrophages (142). However, they could also detect higher levels of mature IL-1β secreted by IKKβ-deficient macrophages, which might be a result of increased CASP1 activation due to enhanced apoptosis (142). Similar to these studies on IKKβ, Zhao. et al. reported that pharmacological suppression of NEMO ubiquitination resulted in reduced *Il1b* and *Nlrp3* expression in LPS-stimulated mouse macrophages (143). Despite scarce evidence, it is tempting to speculate that NEMO deficiency might also result in aberrant NLRP3 inflammasome activation similar to IKKβ. However, since NF-κB signaling controls various central (non-)immune functions, it is hard to differentiate the effects on single effector mechanisms such as inflammasome activation.

#### NOD2 and TRIM22

Nucleotide-binding oligomerization domain 2 (NOD2) is an intracellular pattern recognition receptor (PRR) of the NLR protein family detecting muramyl dipeptide (MDP), which is a component of the bacterial cell wall (81, 144–146). Upon activation, NOD2 signaling induces expression of pro-inflammatory cytokines *via* RIPK2- and NF-κB-mediated signaling and contributes to clearance of different pathogens (144, 146, 147). Of note, genome-wide association studies demonstrated that single nucleotide polymorphisms (SNPs) in *NOD2* represent the strongest genetic risk factor for the development of CD (81, 148, 149). However, mono- or biallelic *NOD2* mutations are not considered as a monogenic cause for IBD, as they can be also frequently found in the genome of healthy humans (81, 150). In contrast, mutations in the NOD2 regulator *tripartite motif containing 22 gene* (*TRIM22*) were shown to cause severe refractory VEO-IBD associated with diarrhea, failure-to-thrive, and multiple infections (151). TRIM22 is a RING finger E3 ubiquitin ligase that catalyzes K63 polyubiquitination of NOD2 and thereby controls NOD2 signaling function (151). Since NOD2 can regulate NF-κB signaling, it is likely that NOD2 signaling may also influence expression of important inflammasome components (i.e., *NLRP3*, *IL1B, IL18*). Indeed, studies in a mouse model of MDP-induced eye inflammation could demonstrate NOD2-mediated production of IL-1β and IL-18 *in vivo* (152). In the human setting, macrophages from CD patients expressing homozygous *NOD2* frameshift mutations fail to induce *IL1B* expression upon MDP stimulation demonstrating a critical role for NOD2 in regulating *IL1B* expression (153). Furthermore, PBMCs from NOD2-deficient CD patients demonstrated a reduced IL-1β secretion in response to MDP/TNF-α co-stimulation indicating that NOD2 signaling also regulates post-translational mechanisms influencing inflammasome activity (153). In line, Hsu et al. demonstrated that MDP and Anthrax toxin stimulation induces formation of the NOD2/NLRP1/CASP1 complex catalyzing IL-1β maturation in mouse macrophages (154). Contrary to MDP stimulation, NOD2 was shown to negatively regulate TLR1/2-mediated induction of *Il1b* expression indicating the complex signaling mechanisms controlled by NOD2 and NF-κB (155). Interestingly, TRIM22 was also shown to support NLRP3 inflammasome responses upon oxygen-glucose deprivation in a neuronal cell line substantiating a potential role of NOD2-mediated signaling on inflammasome activation (109). Similar to the expressivity of NOD2-deficient patients, the role of NOD2 in inflammasome activation and intestinal inflammation models is not completely understood (156, 157). For example, Umiker et al. showed that colitis in *Nod2* knock-out (KO) mice was driven by NLRP3 inflammasome activity, but the underlying mechanisms of increased NLRP3 activity are still unclear (157).

### Cell death regulators

#### CASP8, RIPK1, and XIAP

Patients with X-linked inhibitor of apoptosis (XIAP) deficiency present with a primary immunodeficiency characterized by hemophagocytic lymphohistiocytosis, severe infections, splenomegaly, and cytopenia (113, 158, 159). However, XIAP deficiency was also shown to often manifest with VEO-IBD (113, 158–161). Up to 4% of pediatric IBD has been associated with mutations in XIAP (113, 160, 161). As proposed by its name, XIAP can block apoptosis by inhibiting CASP-3, -7, and -9 *via* baculovirus IAP repeat (BIR) domains (113, 162–164). Furthermore, XIAP was shown to be essential for propagation of NOD2-mediated NF-κB signaling downstream of NOD2 and expression of important NLRP3 inflammasome components (113, 165, 166). In line, cells deficient for XIAP-related signaling components [receptor-interacting protein kinase (RIPK)2, BIRC2, and BIRC3] fail to induce expression of *IL1B* upon exposure to the NOD2 agonist MDP (113, 165, 166). However, loss of XIAP resulted in increased IL-1β secretion and cell death in response to various TLR agonists providing a rationale for autoinflammatory symptoms observed in XIAP deficiency (113–115). Aberrant inflammasome and cell death responses upon loss of XIAP in myeloid cells were shown to be dependent on TNF-, RIPK3-, and CASP8-mediated signaling processes (113–115). In the absence of XIAP, TLR- and TNFR-mediated signaling induces ubiquitination of RIPK1 causing activation of RIPK1 and RIPK3, which results in formation of a complex called ripoptosome that recruits and activates CASP8 (113, 115, 167, 168). Mature CASP8 can induce apoptosis, NLRP3 inflammasome activation, and cleavage of IL-1β demonstrating a direct XIAP-RIPK-CASP8-inflammasome axis (113, 114). Of note, TLR- or TNFR-mediated RIPK3 activation in the absence of CASP8 has been shown to induce NLRP3 inflammasomes and necroptotic cell death (113, 114).

In line with the importance of the XIAP-ripoptosome-CASP8 axis, germline loss-of-function mutations in *RIPK1* and *CASP8* were recently shown to cause VEO-IBD (92, 107, 108). Interestingly, RIPK1 and CASP8 deficiencies resulted in increased premature NLRP3 inflammasome activity characterized by higher IL-1β secretion without requirement of a second signal. Of note, enhanced inflammasome activity was associated with abnormal cell death responses (92, 107, 108). Overall, identification of causative mutations in all these three genes controlling activation of NLRP3 inflammasomes downstream of different immune signaling pathways exemplified the role of inflammasome activation and cell death regulation in IBD pathophysiology and intestinal homeostasis. The only available curative treatment option for VEO-IBD caused by XIAP deficiency is allogeneic HSCT demonstrating the urgency to find treatment alternatives (113, 159, 169). Similarly, there are no curative therapeutics available for RIPK1 or CASP8 deficiencies affecting both the immune system and intestinal epithelium. Since all three genetic defects are characterized by an increased inflammasome activity with higher IL-1β secretion, usage of therapies targeting inflammasomes and/or anti-IL-1R antibodies might represent an attractive approach for treatment.

#### Interleukin-10 receptor

IL-10R deficiency was the first identified monogenic cause for severe VEO-IBD accompanied by perianal disease and folliculitis, which can be only cured by allogeneic HSCT due to the underlying primary immunodeficiency (85, 170). The IL-10 receptor is a heterotetrameric protein complex consisting of two IL-10R1 and IL-10R2 subunits, which are encoded by *IL10RA* and *IL10RB* (171). The corresponding ligand IL-10 is a highly potent anti-inflammatory cytokine controlling pleiotropic functions in the immune systems (171–175). Of note, IL-10-mediated signaling was also shown to inhibit NLRP3 inflammasome activation on a transcriptional and post-translational level (94, 176). In line, IL-10-deficient mice showed increased NLRP3 inflammasome activation and IL-1β levels (94, 95, 177). Enhanced inflammasome activity manifested in mice prior to onset of colitis and the disease could be successfully treated by blockade of NLRP3 inflammasomes or IL-1β signaling demonstrating that symptoms of IL-10 deficiency are mediated by inflammasome perturbation (96, 177, 178). Analogously, cells from IL-10R-deficient patients showed increased and premature NLRP3 inflammasome activation as well as enhanced IL-1β secretion (94–96). Mechanistically, deficient IL-10 signaling was demonstrated to result in altered inflammasome activation by causing defective mitophagy (95). Interestingly, increased IL-1β production in human IL-10R-deficient macrophages can be also caused by alternative inflammasome activation, which is a CASP1-independent process mediated by CASP8 (94). Of note, IL-1β receptor blockade has been shown to ameliorate symptoms in IL-10R-deficient patients providing therapeutic windows for curative allogeneic HSCT (94).

#### WAS

The X-linked Wiskott-Aldrich syndrome (WAS) presents with a life-threatening immunodeficiency characterized by thrombocytopenia and recurrent infections and is caused by mutations in the homonymous gene (179–181). Upon cellular activation, autoinhibition of Wiskott-Aldrich syndrome protein (WASp) is resolved and WASp transfers G-actin to the Arp2/3 complex inducing actin filament formation and branching (181–184). Overall, WASp deficiency has been shown to disturb actin polymerization resulting in impaired chemotactic, migratory, phagocytic, and activation responses of immune cells and platelets (181, 185). Of note, WAS patients can manifest with VEO-IBD and WASp deficiency was shown to cause experimental colitis in mice (110, 186, 187). In fact, intestinal inflammation in *Was* KO mice is driven by macrophages, which develop an inflammatory phenotype characterized by higher levels of pro-inflammatory IL-1β and IL-23 as well as reduced levels of anti-inflammatory IL-10 (110). Analogously, macrophages from WAS patients showed a pro-inflammatory phenotype with higher expression of IL-1β (110). Furthermore, WASp-deficient cells exhibited an increased NLRP3 inflammasome activity, which might be caused by defective clearance of pathogens due to failure of actin assembly around phagocytosed pathogens and defective autophagy (111). Correspondingly, enteropathogen infection of myeloid cells expressing mutant WASp has been shown to enhance ASC speck formation and pyroptosis, indicative of robust inflammasome activation in WASp deficiency (111). Increased inflammasome activation might contribute to autoinflammatory symptoms observed in WAS and might be a target to bridge WAS patients for HSCT, similar to IL-10R-deficiency (110–112). In fact, anti-IL-1R therapy was shown to ameliorate symptoms in one WASp-deficient patient (112). Interestingly, increased inflammasome activation in WASp-deficient cells could be also inhibited by treatment with type I IFNs representing another potential therapeutic option prior to HSCT (111).

#### NADPH complex

Chronic granulomatous disease (CGD) leads to increased susceptibility of recurrent bacterial and fungal infections and is caused by a defective function of the NADPH oxidase complex in innate immune cells (188–190). Interestingly, up to 40% of CGD patients develop intestinal inflammation reminiscent of IBD (191, 192). The NADPH oxidase complex contains gp91-phox, p67-phox, p47-phox, and p22-phox subunits, which are encoded by the genes *CYBB, NCF2, NCF1*, and *CYBA*, respectively (190). Of note, mutations in all four genes have been shown to cause defective production of ROS in innate immune cells resulting in impaired defense against pathogens (190). Production of ROS has been identified as a common intermediate step induced by different inflammasome activators (e.g., ATP, asbestos, silica) and inhibition of ROS generation has been shown to block NLRP3 inflammasome activation (5, 193, 194). Based on these findings defective ROS production might disturb inflammasome activity, however CGD patients show an inflammatory phenotype associated with increased IL-1β release upon TLR stimulation (78, 93). As a potential mechanistic link, De Luca et al. demonstrated that peripheral blood-derived macrophages from NADPH oxidase-deficient mice and CGD patients exhibited defective autophagy resulting in increased IL-1β release (78). Correspondingly, treatment with Anakinra has been shown to enhance a rapid and sustained amelioration of colitis in CGD patients (78).

#### Other immune defects associated with VEO-IBD and altered inflammasome activity

Many inborn errors of immunity are known to present with VEO-IBD, which might be a consequence of the complex interplay between the microbial flora and the immune system at the intestinal barrier. For example, phosphatase and tensin homolog (PTEN) regulates phosphoinositide 3-kinase (PI3K) signaling by dephosphorylating PI(3,4,5)P_3_ and loss-of-function mutations in *PTEN* have been shown to cause autoimmunity or immunodeficiency associated with IBD (195–197). PTEN has been also shown to interact with NLRP3 and KO of PTEN resulted in reduced NLRP3 inflammasome activation after TLR stimulation (106). In detail, PTEN was shown to remove inhibitory phosphorylation from NLRP3 at position Y32, T193, and T195, which enables interaction of NLRP3 with ASC and subsequent oligomerization allowing enhanced inflammasome activation (106). Although data from mouse studies show inflammasome dysregulation in PTEN deficiency, a role of inflammasome activation in human patients with *PTEN* mutations remains to be demonstrated.

The Bruton tyrosine kinase (BTK) is important for B cell receptor (BCR) signaling as well as B cell development and mutations in *BTK* are the most common cause for hypogammaglobulinemia (198–202). Besides its role for B cell development and function, BTK was also shown to interact with NLRP3 and modulate phosphorylation of NLRP3 in myeloid cells (90, 91, 203). Of note, *Btk* KO mice develop severe TNBS-induced colitis, which can be improved by IL-1β blockade indicating a central role of inflammasome activation in BTK-dependent colitis development (91). However, the consequence of BTK activity on human NLRP3 inflammasome activity remains controversial, as reports have shown either increased or decreased NLRP3 inflammasome activation in murine *Btk* KO cells and cells from patients with BTK deficiency (90, 91).

Signal transducer and activator of transcription 1 (STAT1) is a critical signaling molecule in interferon (IFN) responses and STAT1 deficiency causes Mendelian susceptibility to mycobacterial disease associated with severe infections (204, 205). In addition, dominant gain-of-function mutations in STAT1 have been shown to cause a severe immune deficiency associated with polyendocrinopathy and enteropathy (206). Interestingly, STAT1 is also an essential mediator of type I IFN signaling, which can inhibit NLRP1 and NLRP3 inflammasomes (207, 208). Correspondingly, alterations in STAT1 activity and subsequently changed type I IFN responses might predispose patients to dysregulated inflammasome activity upon challenge with pathogens, which still needs to be shown in IBD patients.

Mutations in A disintegrin and metalloprotease 17 (ADAM17) can cause VEO-IBD associated with skin inflammation and susceptibility to gastrointestinal and skin infections (209). ADAM17 has been shown to cleave the pro-inflammatory cytokine TNF-α, which is produced as membrane-bound precursor after activation (210–212). In line, PBMCs from patients with ADAM17 deficiency produce reduced amounts of TNF-α upon LPS stimulation (209). TNF-α-mediated activation of NF-κB signaling has been shown to induce expression of NLRP3 inflammasome components (e.g., NLRP3, IL-1β, and IL-18) and modulate pyrin inflammasome activity (210, 213, 214). In line, targeting TNF-α in a mouse model of autoinflammation caused by NLRP3 mutations was shown to ameliorate symptoms (213). Thus, it is tempting to speculate that failure to produce mature TNF-α in ADAM17 deficiency might also result in disturbed inflammasome activation.

## Conclusion

Several monogenic VEO-IBD defects have been linked to dysregulated inflammasome activity demonstrating the central role of inflammasomes in intestinal homeostasis. As perturbation of inflammasomes can be caused by various genetic entities, studies on monogenic VEO-IBD have highlighted that inflammasomes are controlled by complex regulatory networks and represent a critical common path of human intestinal inflammation. Therefore, targeting inflammasomes and regulatory molecules might be attractive strategies for the treatment of IBD patients. Further studies on the underlying mechanisms in monogenic IBD as disease model will shed light on inflammasome biology and help to identify potential therapeutic targets for rare and common IBD.

## Author contributions

DI and DK wrote the manuscript and prepared the figures. All authors contributed to the article and approved the submitted version.

## Funding

This work has been supported by the Leona M. and Harry B. Helmsley Charitable Trust, DFG (Heinz Maier-Leibnitz-Preis, Collaborative Research Consortium SFB1054 project A05), Else Kröner-Fresenius-Stiftung, Helmholtz Association/Helmholtz Munich (Helmholtz Young Investigator Group), and Care-for-Rare Foundation.

## Conflict of interest

The authors declare that the research was conducted in the absence of any commercial or financial relationships that could be construed as a potential conflict of interest.

## Publisher’s note

All claims expressed in this article are solely those of the authors and do not necessarily represent those of their affiliated organizations, or those of the publisher, the editors and the reviewers. Any product that may be evaluated in this article, or claim that may be made by its manufacturer, is not guaranteed or endorsed by the publisher.

## References

[1] Broz P Dixit VM. Inflammasomes: mechanism of assembly, regulation and signalling. Nat Rev Immunol (2016) 16(7):407–20. doi: 10.1038/nri.2016.58 27291964

[2] Martinon F Burns K Tschopp J. The inflammasome: a molecular platform triggering activation of inflammatory caspases and processing of proIL-beta. Mol Cell (2002) 10(2):417–26. doi: 10.1016/S1097-2765(02)00599-3 12191486

[3] Schroder K Tschopp J. The inflammasomes. Cell (2010) 140(6):821–32. doi: 10.1016/j.cell.2010.01.040 20303873

[4] Strowig T Henao-Mejia J Elinav E Flavell R. Inflammasomes in health and disease. Nature (2012) 481(7381):278–86. doi: 10.1038/nature10759 22258606

[5] Dostert C Petrilli V Van Bruggen R Steele C Mossman BT Tschopp J. Innate immune activation through Nalp3 inflammasome sensing of asbestos and silica. Science (2008) 320(5876):674–7. doi: 10.1126/science.1156995 PMC2396588 18403674

[6] Fernandes-Alnemri T Yu JW Datta P Wu J Alnemri ES. AIM2 activates the inflammasome and cell death in response to cytoplasmic DNA. Nature (2009) 458(7237):509–13. doi: 10.1038/nature07710 PMC2862225 19158676

[7] Halle A Hornung V Petzold GC Stewart CR Monks BG Reinheckel T. The NALP3 inflammasome is involved in the innate immune response to amyloid-beta. Nat Immunol (2008) 9(8):857–65. doi: 10.1038/ni.1636 PMC3101478 18604209

[8] Hornung V Ablasser A Charrel-Dennis M Bauernfeind F Horvath G Caffrey DR. AIM2 recognizes cytosolic dsDNA and forms a caspase-1-activating inflammasome with ASC. Nature (2009) 458(7237):514–8. doi: 10.1038/nature07725 PMC2726264 19158675

[9] Hornung V Bauernfeind F Halle A Samstad EO Kono H Rock KL. Silica crystals and aluminum salts activate the NALP3 inflammasome through phagosomal destabilization. Nat Immunol (2008) 9(8):847–56. doi: 10.1038/ni.1631 PMC2834784 18604214

[10] Hornung V Latz E. Critical functions of priming and lysosomal damage for NLRP3 activation. Eur J Immunol (2010) 40(3):620–3. doi: 10.1002/eji.200940185 PMC3893565 20201015

[11] Petrilli V Papin S Dostert C Mayor A Martinon F Tschopp J. Activation of the NALP3 inflammasome is triggered by low intracellular potassium concentration. Cell Death Differ (2007) 14(9):1583–9. doi: 10.1038/sj.cdd.4402195 17599094

[12] Franchi L Amer A Body-Malapel M Kanneganti TD Ozoren N Jagirdar R. Cytosolic flagellin requires ipaf for activation of caspase-1 and interleukin 1beta in salmonella-infected macrophages. Nat Immunol (2006) 7(6):576–82. doi: 10.1038/ni1346 16648852

[13] Mariathasan S Newton K Monack DM Vucic D French DM Lee WP. Differential activation of the inflammasome by caspase-1 adaptors ASC and ipaf. Nature (2004) 430(6996):213–8. doi: 10.1038/nature02664 15190255

[14] Miao EA Alpuche-Aranda CM Dors M Clark AE Bader MW Miller SI. Cytoplasmic flagellin activates caspase-1 and secretion of interleukin 1beta *via* ipaf. Nat Immunol (2006) 7(6):569–75. doi: 10.1038/ni1344 16648853

[15] Miao EA Mao DP Yudkovsky N Bonneau R Lorang CG Warren SE. Innate immune detection of the type III secretion apparatus through the NLRC4 inflammasome. Proc Natl Acad Sci USA (2010) 107(7):3076–80. doi: 10.1073/pnas.0913087107 PMC2840275 20133635

[16] Man SM. Inflammasomes in the gastrointestinal tract: infection, cancer and gut microbiota homeostasis. Nat Rev Gastroenterol Hepatol (2018) 15(12):721–37. doi: 10.1038/s41575-018-0054-1 PMC7097092 30185915

[17] Broz P von Moltke J Jones JW Vance RE Monack DM. Differential requirement for caspase-1 autoproteolysis in pathogen-induced cell death and cytokine processing. Cell Host Microbe (2010) 8(6):471–83. doi: 10.1016/j.chom.2010.11.007 PMC3016200 21147462

[18] Guey B Bodnar M Manie SN Tardivel A Petrilli V. Caspase-1 autoproteolysis is differentially required for NLRP1b and NLRP3 inflammasome function. Proc Natl Acad Sci USA (2014) 111(48):17254–9. doi: 10.1073/pnas.1415756111 PMC4260594 25404286

[19] Poyet JL Srinivasula SM Tnani M Razmara M Fernandes-Alnemri T Alnemri ES. Identification of ipaf, a human caspase-1-activating protein related to apaf-1. J Biol Chem (2001) 276(30):28309–13. doi: 10.1074/jbc.C100250200 11390368

[20] Agostini L Martinon F Burns K McDermott MF Hawkins PN Tschopp J. NALP3 forms an IL-1beta-processing inflammasome with increased activity in muckle-wells autoinflammatory disorder. Immunity (2004) 20(3):319–25. doi: 10.1016/S1074-7613(04)00046-9 15030775

[21] Lu A Magupalli VG Ruan J Yin Q Atianand MK Vos MR. Unified polymerization mechanism for the assembly of ASC-dependent inflammasomes. Cell (2014) 156(6):1193–206. doi: 10.1016/j.cell.2014.02.008 PMC4000066 24630722

[22] Richards N Schaner P Diaz A Stuckey J Shelden E Wadhwa A. Interaction between pyrin and the apoptotic speck protein (ASC) modulates ASC-induced apoptosis. J Biol Chem (2001) 276(42):39320–9. doi: 10.1074/jbc.M104730200 11498534

[23] Thornberry NA Bull HG Calaycay JR Chapman KT Howard AD Kostura MJ. A novel heterodimeric cysteine protease is required for interleukin-1 beta processing in monocytes. Nature (1992) 356(6372):768–74. doi: 10.1038/356768a0 1574116

[24] Boucher D Monteleone M Coll RC Chen KW Ross CM Teo JL. Caspase-1 self-cleavage is an intrinsic mechanism to terminate inflammasome activity. J Exp Med (2018) 215(3):827–40. doi: 10.1084/jem.20172222 PMC5839769 29432122

[25] He WT Wan H Hu L Chen P Wang X Huang Z. Gasdermin d is an executor of pyroptosis and required for interleukin-1beta secretion. Cell Res (2015) 25(12):1285–98. doi: 10.1038/cr.2015.139 PMC4670995 26611636

[26] Shi J Zhao Y Wang K Shi X Wang Y Huang H. Cleavage of GSDMD by inflammatory caspases determines pyroptotic cell death. Nature (2015) 526(7575):660–5. doi: 10.1038/nature15514 26375003

[27] Dinarello CA. Interleukin-1 beta, interleukin-18, and the interleukin-1 beta converting enzyme. Ann N Y Acad Sci (1998) 856:1–11. doi: 10.1111/j.1749-6632.1998.tb08307.x 9917859

[28] Ghayur T Banerjee S Hugunin M Butler D Herzog L Carter A. Caspase-1 processes IFN-gamma-inducing factor and regulates LPS-induced IFN-gamma production. Nature (1997) 386(6625):619–23. doi: 10.1038/386619a0 9121587

[29] Gu Y Kuida K Tsutsui H Ku G Hsiao K Fleming MA. Activation of interferon-gamma inducing factor mediated by interleukin-1beta converting enzyme. Science (1997) 275(5297):206–9. doi: 10.1126/science.275.5297.206 8999548

[30] Bauernfeind FG Horvath G Stutz A Alnemri ES MacDonald K Speert D. Cutting edge: NF-kappaB activating pattern recognition and cytokine receptors license NLRP3 inflammasome activation by regulating NLRP3 expression. J Immunol (2009) 183(2):787–91. doi: 10.4049/jimmunol.0901363 PMC2824855 19570822

[31] Zhen Y Zhang H. NLRP3 inflammasome and inflammatory bowel disease. Front Immunol (2019) 10:276. doi: 10.3389/fimmu.2019.00276 30873162 PMC6403142

[32] Juliana C Fernandes-Alnemri T Kang S Farias A Qin F Alnemri ES. Non-transcriptional priming and deubiquitination regulate NLRP3 inflammasome activation. J Biol Chem (2012) 287(43):36617–22. doi: 10.1074/jbc.M112.407130 PMC3476327 22948162

[33] Py BF Kim MS Vakifahmetoglu-Norberg H Yuan J. Deubiquitination of NLRP3 by BRCC3 critically regulates inflammasome activity. Mol Cell (2013) 49(2):331–8. doi: 10.1016/j.molcel.2012.11.009 23246432

[34] Mishra BB Rathinam VA Martens GW Martinot AJ Kornfeld H Fitzgerald KA. Nitric oxide controls the immunopathology of tuberculosis by inhibiting NLRP3 inflammasome-dependent processing of IL-1beta. Nat Immunol (2013) 14(1):52–60. doi: 10.1038/ni.2474 23160153 PMC3721324

[35] Hara H Tsuchiya K Kawamura I Fang R Hernandez-Cuellar E Shen Y. Phosphorylation of the adaptor ASC acts as a molecular switch that controls the formation of speck-like aggregates and inflammasome activity. Nat Immunol (2013) 14(12):1247–55. doi: 10.1038/ni.2749 PMC4813763 24185614

[36] Qu Y Misaghi S Izrael-Tomasevic A Newton K Gilmour LL Lamkanfi M. Phosphorylation of NLRC4 is critical for inflammasome activation. Nature (2012) 490(7421):539–42. doi: 10.1038/nature11429 22885697

[37] Gao W Yang J Liu W Wang Y Shao F. Site-specific phosphorylation and microtubule dynamics control pyrin inflammasome activation. Proc Natl Acad Sci USA (2016) 113(33):E4857–66. doi: 10.1073/pnas.1601700113 PMC4995971 27482109

[38] Schnappauf O Chae JJ Kastner DL Aksentijevich I. The pyrin inflammasome in health and disease. Front Immunol (2019) 10:1745. doi: 10.3389/fimmu.2019.01745 31456795 PMC6698799

[39] Lei-Leston AC Murphy AG Maloy KJ. Epithelial cell inflammasomes in intestinal immunity and inflammation. Front Immunol (2017) 8:1168. doi: 10.3389/fimmu.2017.01168 28979266 PMC5611393

[40] Jung HC Eckmann L Yang SK Panja A Fierer J Morzycka-Wroblewska E. A distinct array of proinflammatory cytokines is expressed in human colon epithelial cells in response to bacterial invasion. J Clin Invest (1995) 95(1):55–65. doi: 10.1172/JCI117676 7814646 PMC295369

[41] Knodler LA Crowley SM Sham HP Yang H Wrande M Ma C. Noncanonical inflammasome activation of caspase-4/caspase-11 mediates epithelial defenses against enteric bacterial pathogens. Cell Host Microbe (2014) 16(2):249–56. doi: 10.1016/j.chom.2014.07.002 PMC4157630 25121752

[42] Thinwa J Segovia JA Bose S Dube PH. Integrin-mediated first signal for inflammasome activation in intestinal epithelial cells. J Immunol (2014) 193(3):1373–82. doi: 10.4049/jimmunol.1400145 PMC4174679 24965773

[43] Bazan JF Timans JC Kastelein RA. A newly defined interleukin-1? Nature (1996) 379(6566):591. doi: 10.1038/379591a0 8628393

[44] Okamura H Tsutsi H Komatsu T Yutsudo M Hakura A Tanimoto T. Cloning of a new cytokine that induces IFN-gamma production by T cells. Nature (1995) 378(6552):88–91. doi: 10.1038/378088a0 7477296

[45] Wang P Zhu S Yang L Cui S Pan W Jackson R. Nlrp6 regulates intestinal antiviral innate immunity. Science (2015) 350(6262):826–30. doi: 10.1126/science.aab3145 PMC4927078 26494172

[46] Rauch I Deets KA Ji DX von Moltke J Tenthorey JL Lee AY. NAIP-NLRC4 inflammasomes coordinate intestinal epithelial cell expulsion with eicosanoid and IL-18 release *via* activation of caspase-1 and -8. Immunity (2017) 46(4):649–59. doi: 10.1016/j.immuni.2017.03.016 PMC5476318 28410991

[47] Sellin ME Muller AA Felmy B Dolowschiak T Diard M Tardivel A. Epithelium-intrinsic NAIP/NLRC4 inflammasome drives infected enterocyte expulsion to restrict salmonella replication in the intestinal mucosa. Cell Host Microbe (2014) 16(2):237–48. doi: 10.1016/j.chom.2014.07.001 25121751

[48] Wlodarska M Thaiss CA Nowarski R Henao-Mejia J Zhang JP Brown EM. NLRP6 inflammasome orchestrates the colonic host-microbial interface by regulating goblet cell mucus secretion. Cell (2014) 156(5):1045–59. doi: 10.1016/j.cell.2014.01.026 PMC4017640 24581500

[49] Acosta-Rodriguez EV Napolitani G Lanzavecchia A Sallusto F. Interleukins 1beta and 6 but not transforming growth factor-beta are essential for the differentiation of interleukin 17-producing human T helper cells. Nat Immunol (2007) 8(9):942–9. doi: 10.1038/ni1496 17676045

[50] Coccia M Harrison OJ Schiering C Asquith MJ Becher B Powrie F. IL-1beta mediates chronic intestinal inflammation by promoting the accumulation of IL-17A secreting innate lymphoid cells and CD4(+) Th17 cells. J Exp Med (2012) 209(9):1595–609. doi: 10.1084/jem.20111453 PMC3428945 22891275

[51] Al-Sadi RM Ma TY. IL-1beta causes an increase in intestinal epithelial tight junction permeability. J Immunol (2007) 178(7):4641–9. doi: 10.4049/jimmunol.178.7.4641 PMC3724221 17372023

[52] Cox CB Storm EE Kapoor VN Chavarria-Smith J Lin DL Wang L. IL-1R1-dependent signaling coordinates epithelial regeneration in response to intestinal damage. Sci Immunol (2021) 6(59):eabe8856. doi: 10.1126/sciimmunol.abe8856 33963061

[53] Mascarenhas JO Goodrich ME Eichelberger H McGee DW. Polarized secretion of IL-6 by IEC-6 intestinal epithelial cells: differential effects of IL-1 beta and TNF-alpha. Immunol Invest (1996) 25(4):333–40. doi: 10.3109/08820139609059315 8805054

[54] Yan SR Joseph RR Wang J Stadnyk AW. Differential pattern of inflammatory molecule regulation in intestinal epithelial cells stimulated with IL-1. J Immunol (2006) 177(8):5604–11. doi: 10.4049/jimmunol.177.8.5604 17015748

[55] Miao EA Leaf IA Treuting PM Mao DP Dors M Sarkar A. Caspase-1-induced pyroptosis is an innate immune effector mechanism against intracellular bacteria. Nat Immunol (2010) 11(12):1136–42. doi: 10.1038/ni.1960 PMC3058225 21057511

[56] Franchi L Kamada N Nakamura Y Burberry A Kuffa P Suzuki S. NLRC4-driven production of IL-1beta discriminates between pathogenic and commensal bacteria and promotes host intestinal defense. Nat Immunol (2012) 13(5):449–56. doi: 10.1038/ni.2263 PMC3361590 22484733

[57] Kim DJ Park JH Franchi L Backert S Nunez G. The cag pathogenicity island and interaction between TLR2/NOD2 and NLRP3 regulate IL-1beta production in helicobacter pylori infected dendritic cells. Eur J Immunol (2013) 43(10):2650–8. doi: 10.1002/eji.201243281 PMC3797179 23818043

[58] Perez-Figueroa E Torres J Sanchez-Zauco N Contreras-Ramos A Alvarez-Arellano L Maldonado-Bernal C. Activation of NLRP3 inflammasome in human neutrophils by helicobacter pylori infection. Innate Immun (2016) 22(2):103–12. doi: 10.1177/1753425915619475 26610398

[59] Semper RP Mejias-Luque R Gross C Anderl F Muller A Vieth M. Helicobacter pylori-induced IL-1beta secretion in innate immune cells is regulated by the NLRP3 inflammasome and requires the cag pathogenicity island. J Immunol (2014) 193(7):3566–76. doi: 10.4049/jimmunol.1400362 25172489

[60] Barlan AU Griffin TM McGuire KA Wiethoff CM. Adenovirus membrane penetration activates the NLRP3 inflammasome. J Virol (2011) 85(1):146–55. doi: 10.1128/JVI.01265-10 PMC3014182 20980503

[61] Li Z Wang H Chen Y Niu J Guo Q Leng Q. Interleukin-18 protects mice from enterovirus 71 infection. Cytokine (2017) 96:132–7. doi: 10.1016/j.cyto.2017.04.002 28399485

[62] Muruve DA Petrilli V Zaiss AK White LR Clark SA Ross PJ. The inflammasome recognizes cytosolic microbial and host DNA and triggers an innate immune response. Nature (2008) 452(7183):103–7. doi: 10.1038/nature06664 18288107

[63] Wang H Lei X Xiao X Yang C Lu W Huang Z. Reciprocal regulation between enterovirus 71 and the NLRP3 inflammasome. Cell Rep (2015) 12(1):42–8. doi: 10.1016/j.celrep.2015.05.047 26119741

[64] Reyes Ruiz VM Ramirez J Naseer N Palacio NM Siddarthan IJ Yan BM. Broad detection of bacterial type III secretion system and flagellin proteins by the human NAIP/NLRC4 inflammasome. Proc Natl Acad Sci USA (2017) 114(50):13242–7. doi: 10.1073/pnas.1710433114 PMC5740664 29180436

[65] Xu H Yang J Gao W Li L Li P Zhang L. Innate immune sensing of bacterial modifications of rho GTPases by the pyrin inflammasome. Nature (2014) 513(7517):237–41. doi: 10.1038/nature13449 24919149

[66] Ng J Hirota SA Gross O Li Y Ulke-Lemee A Potentier MS. Clostridium difficile toxin-induced inflammation and intestinal injury are mediated by the inflammasome. Gastroenterology (2010) 139(2):542–52,52.e1-3. doi: 10.1053/j.gastro.2010.04.005 20398664

[67] Man SM Ekpenyong A Tourlomousis P Achouri S Cammarota E Hughes K. Actin polymerization as a key innate immune effector mechanism to control salmonella infection. Proc Natl Acad Sci USA (2014) 111(49):17588–93. doi: 10.1073/pnas.1419925111 PMC4267384 25422455

[68] Ligumsky M Simon PL Karmeli F Rachmilewitz D. Role of interleukin 1 in inflammatory bowel disease–enhanced production during active disease. Gut (1990) 31(6):686–9. doi: 10.1136/gut.31.6.686 PMC1378497 2379873

[69] Lazaridis LD Pistiki A Giamarellos-Bourboulis EJ Georgitsi M Damoraki G Polymeros D. Activation of NLRP3 inflammasome in inflammatory bowel disease: Differences between crohn's disease and ulcerative colitis. Dig Dis Sci (2017) 62(9):2348–56. doi: 10.1007/s10620-017-4609-8 28523573

[70] Monteleone G Trapasso F Parrello T Biancone L Stella A Iuliano R. Bioactive IL-18 expression is up-regulated in crohn's disease. J Immunol (1999) 163(1):143–7. doi: 10.4049/jimmunol.163.1.143 10384110

[71] Pizarro TT Michie MH Bentz M Woraratanadharm J Smith MF Jr. Foley E. IL-18, a novel immunoregulatory cytokine, is up-regulated in crohn's disease: expression and localization in intestinal mucosal cells. J Immunol (1999) 162(11):6829–35. doi: 10.4049/jimmunol.162.11.6829 10352304

[72] Mitsialis V Wall S Liu P Ordovas-Montanes J Parmet T Vukovic M. Single-cell analyses of colon and blood reveal distinct immune cell signatures of ulcerative colitis and crohn's disease. Gastroenterology (2020) 159(2):591–608.e10. doi: 10.1053/j.gastro.2020.04.074 32428507 PMC8166295

[73] Liso M Verna G Cavalcanti E De Santis S Armentano R Tafaro A. Interleukin 1beta blockade reduces intestinal inflammation in a murine model of tumor necrosis factor-independent ulcerative colitis. Cell Mol Gastroenterol Hepatol (2022) 14(1):151–71. doi: 10.1016/j.jcmgh.2022.03.003 PMC9120241 35314399

[74] Gao SJ Zhang L Lu W Wang L Chen L Zhu Z. Interleukin-18 genetic polymorphisms contribute differentially to the susceptibility to crohn's disease. World J Gastroenterol (2015) 21(28):8711–22. doi: 10.3748/wjg.v21.i28.8711 PMC4515852 26229413

[75] Schoultz I Verma D Halfvarsson J Torkvist L Fredrikson M Sjoqvist U. Combined polymorphisms in genes encoding the inflammasome components NALP3 and CARD8 confer susceptibility to crohn's disease in Swedish men. Am J Gastroenterol (2009) 104(5):1180–8. doi: 10.1038/ajg.2009.29 19319132

[76] Villani AC Lemire M Fortin G Louis E Silverberg MS Collette C. Common variants in the NLRP3 region contribute to crohn's disease susceptibility. Nat Genet (2009) 41(1):71–6. doi: 10.1038/ng.285 PMC2728932 19098911

[77] Mao L Kitani A Similuk M Oler AJ Albenberg L Kelsen J. Loss-of-function CARD8 mutation causes NLRP3 inflammasome activation and crohn's disease. J Clin Invest (2018) 128(5):1793–806. doi: 10.1172/JCI98642 PMC5919822 29408806

[78] de Luca A Smeekens SP Casagrande A Iannitti R Conway KL Gresnigt MS. IL-1 receptor blockade restores autophagy and reduces inflammation in chronic granulomatous disease in mice and in humans. Proc Natl Acad Sci USA (2014) 111(9):3526–31. doi: 10.1073/pnas.1322831111 PMC3948220 24550444

[79] Ten Hove T Corbaz A Amitai H Aloni S Belzer I Graber P. Blockade of endogenous IL-18 ameliorates TNBS-induced colitis by decreasing local TNF-alpha production in mice. Gastroenterology (2001) 121(6):1372–9. doi: 10.1053/gast.2001.29579 11729116

[80] Thomas MG Bayliss C Bond S Dowling F Galea J Jairath V. Trial summary and protocol for a phase II randomised placebo-controlled double-blinded trial of interleukin 1 blockade in acute severe colitis: the IASO trial. BMJ Open (2019) 9(2):e023765. doi: 10.1136/bmjopen-2018-023765 PMC6398753 30772849

[81] Abraham C Cho JH. Inflammatory bowel disease. N Engl J Med (2009) 361(21):2066–78. doi: 10.1056/NEJMra0804647 PMC3491806 19923578

[82] Heyman MB Kirschner BS Gold BD Ferry G Baldassano R Cohen SA. Children with early-onset inflammatory bowel disease (IBD): analysis of a pediatric IBD consortium registry. J Pediatr (2005) 146(1):35–40. doi: 10.1016/j.jpeds.2004.08.043 15644819

[83] Uhlig HH Charbit-Henrion F Kotlarz D Shouval DS Schwerd T Strisciuglio C. Clinical genomics for the diagnosis of monogenic forms of inflammatory bowel disease: A position paper from the paediatric IBD Porto group of European society of paediatric gastroenterology, hepatology and nutrition. J Pediatr Gastroenterol Nutr (2021) 72(3):456–73. doi: 10.1097/MPG.0000000000003017 PMC8221730 33346580

[84] Uhlig HH Schwerd T Koletzko S Shah N Kammermeier J Elkadri A. The diagnostic approach to monogenic very early onset inflammatory bowel disease. Gastroenterology (2014) 147(5):990–1007.e3. doi: 10.1053/j.gastro.2014.07.023 25058236 PMC5376484

[85] Kotlarz D Beier R Murugan D Diestelhorst J Jensen O Boztug K. Loss of interleukin-10 signaling and infantile inflammatory bowel disease: implications for diagnosis and therapy. Gastroenterology (2012) 143(2):347–55. doi: 10.1053/j.gastro.2012.04.045 22549091

[86] Ouahed J Spencer E Kotlarz D Shouval DS Kowalik M Peng K. Very early onset inflammatory bowel disease: A clinical approach with a focus on the role of genetics and underlying immune deficiencies. Inflammation Bowel Dis (2020) 26(6):820–42. doi: 10.1093/ibd/izz259 PMC7216773 31833544

[87] Pazmandi J Kalinichenko A Ardy RC Boztug K. Early-onset inflammatory bowel disease as a model disease to identify key regulators of immune homeostasis mechanisms. Immunol Rev (2019) 287(1):162–85. doi: 10.1111/imr.12726 PMC7379380 30565237

[88] Sullivan KE Conrad M Kelsen JR. Very early-onset inflammatory bowel disease: an integrated approach. Curr Opin Allergy Clin Immunol (2018) 18(6):459–69. doi: 10.1097/ACI.0000000000000484 PMC9082279 30299395

[89] Uhlig HH Muise AM. Clinical genomics in inflammatory bowel disease. Trends Genet (2017) 33(9):629–41. doi: 10.1016/j.tig.2017.06.008 28755896

[90] Bittner ZA Liu X Mateo Tortola M Tapia-Abellan A Shankar S Andreeva L. BTK operates a phospho-tyrosine switch to regulate NLRP3 inflammasome activity. J Exp Med (2021) 218(11):e20201656. doi: 10.1084/jem.20201656 34554188 PMC8480672

[91] Mao L Kitani A Hiejima E Montgomery-Recht K Zhou W Fuss I. Bruton tyrosine kinase deficiency augments NLRP3 inflammasome activation and causes IL-1beta-mediated colitis. J Clin Invest (2020) 130(4):1793–807. doi: 10.1172/JCI128322 PMC7108929 31895698

[92] Lehle AS Farin HF Marquardt B Michels BE Magg T Li Y. Intestinal inflammation and dysregulated immunity in patients with inherited caspase-8 deficiency. Gastroenterology (2019) 156(1):275–8. doi: 10.1053/j.gastro.2018.09.041 30267714

[93] Meissner F Seger RA Moshous D Fischer A Reichenbach J Zychlinsky A. Inflammasome activation in NADPH oxidase defective mononuclear phagocytes from patients with chronic granulomatous disease. Blood (2010) 116(9):1570–3. doi: 10.1182/blood-2010-01-264218 PMC2938844 20495074

[94] Shouval DS Biswas A Kang YH Griffith AE Konnikova L Mascanfroni ID. Interleukin 1beta mediates intestinal inflammation in mice and patients with interleukin 10 receptor deficiency. Gastroenterology (2016) 151(6):1100–4. doi: 10.1053/j.gastro.2016.08.055 PMC5124405 27693323

[95] Ip WKE Hoshi N Shouval DS Snapper S Medzhitov R. Anti-inflammatory effect of IL-10 mediated by metabolic reprogramming of macrophages. Science (2017) 356(6337):513–9. doi: 10.1126/science.aal3535 PMC6260791 28473584

[96] Liu L Dong Y Ye M Jin S Yang J Joosse ME. The pathogenic role of NLRP3 inflammasome activation in inflammatory bowel diseases of both mice and humans. J Crohns Colitis (2017) 11(6):737–50. doi: 10.1093/ecco-jcc/jjw219 PMC5881697 27993998

[97] Park YH Wood G Kastner DL Chae JJ. Pyrin inflammasome activation and RhoA signaling in the autoinflammatory diseases FMF and HIDS. Nat Immunol (2016) 17(8):914–21. doi: 10.1038/ni.3457 PMC4955684 27270401

[98] Migita K Izumi Y Fujikawa K Agematsu K Masumoto J Jiuchi Y. Dysregulated mature IL-1beta production in familial Mediterranean fever. Rheumatol (Oxford) (2015) 54(4):660–5. doi: 10.1093/rheumatology/keu359 25240611

[99] Ozen S Demirkaya E Erer B Livneh A Ben-Chetrit E Giancane G. EULAR recommendations for the management of familial Mediterranean fever. Ann Rheum Dis (2016) 75(4):644–51. doi: 10.1136/annrheumdis-2015-208690 26802180

[100] Roldan R Ruiz AM Miranda MD Collantes E. Anakinra: new therapeutic approach in children with familial Mediterranean fever resistant to colchicine. Joint Bone Spine (2008) 75(4):504–5. doi: 10.1016/j.jbspin.2008.04.001 18541452

[101] Politiek FA Waterham HR. Compromised protein prenylation as pathogenic mechanism in mevalonate kinase deficiency. Front Immunol (2021) 12:724991. doi: 10.3389/fimmu.2021.724991 34539662 PMC8446354

[102] Levy M Arion A Berrebi D Cuisset L Jeanne-Pasquier C Bader-Meunier B. Severe early-onset colitis revealing mevalonate kinase deficiency. Pediatrics (2013) 132(3):e779–83. doi: 10.1542/peds.2012-3344 23979089

[103] Canna SW de Jesus AA Gouni S Brooks SR Marrero B Liu Y. An activating NLRC4 inflammasome mutation causes autoinflammation with recurrent macrophage activation syndrome. Nat Genet (2014) 46(10):1140–6. doi: 10.1038/ng.3089 PMC4177369 25217959

[104] Romberg N Al Moussawi K Nelson-Williams C Stiegler AL Loring E Choi M. Mutation of NLRC4 causes a syndrome of enterocolitis and autoinflammation. Nat Genet (2014) 46(10):1135–9. doi: 10.1038/ng.3066 PMC4177367 25217960

[105] Canna SW Girard C Malle L de Jesus A Romberg N Kelsen J. Life-threatening NLRC4-associated hyperinflammation successfully treated with IL-18 inhibition. J Allergy Clin Immunol (2017) 139(5):1698–701. doi: 10.1016/j.jaci.2016.10.022 PMC5846100 27876626

[106] Huang Y Wang H Hao Y Lin H Dong M Ye J. Myeloid PTEN promotes chemotherapy-induced NLRP3-inflammasome activation and antitumour immunity. Nat Cell Biol (2020) 22(6):716–27. doi: 10.1038/s41556-020-0510-3 32367047

[107] Li Y Fuhrer M Bahrami E Socha P Klaudel-Dreszler M Bouzidi A. Human RIPK1 deficiency causes combined immunodeficiency and inflammatory bowel diseases. Proc Natl Acad Sci USA (2019) 116(3):970–5. doi: 10.1073/pnas.1813582116 PMC6338855 30591564

[108] Cuchet-Lourenco D Eletto D Wu C Plagnol V Papapietro O Curtis J. Biallelic RIPK1 mutations in humans cause severe immunodeficiency, arthritis, and intestinal inflammation. Science (2018) 361(6404):810–3. doi: 10.1126/science.aar2641 PMC6529353 30026316

[109] Kang C Lu Z Zhu G Chen Y Wu Y. Knockdown of TRIM22 relieves oxygen-glucose Deprivation/Reoxygenation-induced apoptosis and inflammation through inhibition of NF-kappaB/NLRP3 axis. Cell Mol Neurobiol (2021) 41(2):341–51. doi: 10.1007/s10571-020-00855-w PMC11448583 32335773

[110] Biswas A Shouval DS Griffith A Goettel JA Field M Kang YH. WASP-mediated regulation of anti-inflammatory macrophages is IL-10 dependent and is critical for intestinal homeostasis. Nat Commun (2018) 9(1):1779. doi: 10.1038/s41467-018-03670-6 29725003 PMC5934380

[111] Lee PP Lobato-Marquez D Pramanik N Sirianni A Daza-Cajigal V Rivers E. Wiskott-Aldrich syndrome protein regulates autophagy and inflammasome activity in innate immune cells. Nat Commun (2017) 8(1):1576. doi: 10.1038/s41467-017-01676-0 29146903 PMC5691069

[112] Brigida I Scaramuzza S Lazarevic D Cittaro D Ferrua F Leonardelli L. A novel genomic inversion in wiskott-aldrich-associated autoinflammation. J Allergy Clin Immunol (2016) 138(2):619–22.e7. doi: 10.1016/j.jaci.2016.03.007 27113846 PMC4969072

[113] Mudde ACA Booth C Marsh RA. Evolution of our understanding of XIAP deficiency. Front Pediatr (2021) 9:660520. doi: 10.3389/fped.2021.660520 34222142 PMC8247594

[114] Lawlor KE Feltham R Yabal M Conos SA Chen KW Ziehe S. XIAP loss triggers RIPK3- and caspase-8-Driven IL-1beta activation and cell death as a consequence of TLR-MyD88-Induced cIAP1-TRAF2 degradation. Cell Rep (2017) 20(3):668–82. doi: 10.1016/j.celrep.2017.06.073 28723569

[115] Yabal M Muller N Adler H Knies N Gross CJ Damgaard RB. XIAP restricts TNF- and RIP3-dependent cell death and inflammasome activation. Cell Rep (2014) 7(6):1796–808. doi: 10.1016/j.celrep.2014.05.008 24882010

[116] Steiner A Reygaerts T Pontillo A Ceccherini I Moecking J Moghaddas F. Recessive NLRC4-autoinflammatory disease reveals an ulcerative colitis locus. J Clin Immunol (2022) 42(2):325–35. doi: 10.1007/s10875-021-01175-4 PMC8821057 34783940

[117] The international FMF consortium Ancient missense mutations in a new member of the RoRet gene family are likely to cause familial Mediterranean fever. the international FMF consortium. Cell (1997) 90(4):797–807. doi: 10.1016/s0092-8674(00)80539-5 9288758

[118] French FMFC. A candidate gene for familial Mediterranean fever. Nat Genet (1997) 17(1):25–31. doi: 10.1038/ng0997-25 9288094

[119] Yu JW Wu J Zhang Z Datta P Ibrahimi I Taniguchi S. Cryopyrin and pyrin activate caspase-1, but not NF-kappaB, *via* ASC oligomerization. Cell Death Differ (2006) 13(2):236–49. doi: 10.1038/sj.cdd.4401734 16037825

[120] Demirkaya E Erer B Ozen S Ben-Chetrit E. Efficacy and safety of treatments in familial Mediterranean fever: a systematic review. Rheumatol Int (2016) 36(3):325–31. doi: 10.1007/s00296-015-3408-9 26687683

[121] Goldfinger SE. Colchicine for familial Mediterranean fever. N Engl J Med (1972) 287(25):1302. doi: 10.1056/NEJM197212212872514 4636899

[122] Kallinich T Haffner D Niehues T Huss K Lainka E Neudorf U. Colchicine use in children and adolescents with familial Mediterranean fever: literature review and consensus statement. Pediatrics (2007) 119(2):e474–83. doi: 10.1542/peds.2006-1434 17242135

[123] Miziorko HM. Enzymes of the mevalonate pathway of isoprenoid biosynthesis. Arch Biochem Biophys (2011) 505(2):131–43. doi: 10.1016/j.abb.2010.09.028 PMC3026612 20932952

[124] Drenth JP Cuisset L Grateau G Vasseur C van de Velde-Visser SD de Jong JG. Mutations in the gene encoding mevalonate kinase cause hyper-IgD and periodic fever syndrome. international hyper-IgD study group. Nat Genet (1999) 22(2):178–81. doi: 10.1038/9696 10369262

[125] Houten SM Kuis W Duran M de Koning TJ van Royen-Kerkhof A Romeijn GJ. Mutations in MVK, encoding mevalonate kinase, cause hyperimmunoglobulinaemia d and periodic fever syndrome. Nat Genet (1999) 22(2):175–7. doi: 10.1038/9691 10369261

[126] Hoffmann G Gibson KM Brandt IK Bader PI Wappner RS Sweetman L. Mevalonic aciduria–an inborn error of cholesterol and nonsterol isoprene biosynthesis. N Engl J Med (1986) 314(25):1610–4. doi: 10.1056/NEJM198606193142504 3012338

[127] Baldwin AS Jr. The NF-kappa b and I kappa b proteins: new discoveries and insights. Annu Rev Immunol (1996) 14:649–83. doi: 10.1146/annurev.immunol.14.1.649 8717528

[128] May MJ Ghosh S. Signal transduction through NF-kappa b. Immunol Today (1998) 19(2):80–8. doi: 10.1016/S0167-5699(97)01197-3 9509763

[129] Yamaoka S Courtois G Bessia C Whiteside ST Weil R Agou F. Complementation cloning of NEMO, a component of the IkappaB kinase complex essential for NF-kappaB activation. Cell (1998) 93(7):1231–40. doi: 10.1016/S0092-8674(00)81466-X 9657155

[130] Baeuerle PA Baltimore D. I Kappa b: a specific inhibitor of the NF-kappa b transcription factor. Science (1988) 242(4878):540–6. doi: 10.1126/science.3140380 3140380

[131] Beg AA Baldwin AS Jr. The I kappa b proteins: multifunctional regulators of Rel/NF-kappa b transcription factors. Genes Dev (1993) 7(11):2064–70. doi: 10.1101/gad.7.11.2064 8224838

[132] DiDonato JA Hayakawa M Rothwarf DM Zandi E Karin M. A cytokine-responsive IkappaB kinase that activates the transcription factor NF-kappaB. Nature (1997) 388(6642):548–54. doi: 10.1038/41493 9252186

[133] Mercurio F Zhu H Murray BW Shevchenko A Bennett BL Li J. IKK-1 and IKK-2: cytokine-activated IkappaB kinases essential for NF-kappaB activation. Science (1997) 278(5339):860–6. doi: 10.1126/science.278.5339.860 9346484

[134] Regnier CH Song HY Gao X Goeddel DV Cao Z Rothe M. Identification and characterization of an IkappaB kinase. Cell (1997) 90(2):373–83. doi: 10.1016/S0092-8674(00)80344-X 9244310

[135] Rothwarf DM Zandi E Natoli G Karin M. IKK-gamma is an essential regulatory subunit of the IkappaB kinase complex. Nature (1998) 395(6699):297–300. doi: 10.1038/26261 9751060

[136] Zandi E Rothwarf DM Delhase M Hayakawa M Karin M. The IkappaB kinase complex (IKK) contains two kinase subunits, IKKalpha and IKKbeta, necessary for IkappaB phosphorylation and NF-kappaB activation. Cell (1997) 91(2):243–52. doi: 10.1016/S0092-8674(00)80406-7 9346241

[137] Hanson EP Monaco-Shawver L Solt LA Madge LA Banerjee PP May MJ. Hypomorphic nuclear factor-kappaB essential modulator mutation database and reconstitution system identifies phenotypic and immunologic diversity. J Allergy Clin Immunol (2008) 122(6):1169–77.e16. doi: 10.1016/j.jaci.2008.08.018 18851874 PMC2710968

[138] Klemann C Pannicke U Morris-Rosendahl DJ Vlantis K Rizzi M Uhlig H. Transplantation from a symptomatic carrier sister restores host defenses but does not prevent colitis in NEMO deficiency. Clin Immunol (2016) 164:52–6. doi: 10.1016/j.clim.2016.01.010 PMC6101191 26812624

[139] Zonana J Elder ME Schneider LC Orlow SJ Moss C Golabi M. A novel X-linked disorder of immune deficiency and hypohidrotic ectodermal dysplasia is allelic to incontinentia pigmenti and due to mutations in IKK-gamma (NEMO). Am J Hum Genet (2000) 67(6):1555–62. doi: 10.1086/316914 PMC1287930 11047757

[140] Nenci A Becker C Wullaert A Gareus R van Loo G Danese S. Epithelial NEMO links innate immunity to chronic intestinal inflammation. Nature (2007) 446(7135):557–61. doi: 10.1038/nature05698 17361131

[141] Vlantis K Wullaert A Polykratis A Kondylis V Dannappel M Schwarzer R. NEMO prevents RIP kinase 1-mediated epithelial cell death and chronic intestinal inflammation by NF-kappaB-Dependent and -independent functions. Immunity (2016) 44(3):553–67. doi: 10.1016/j.immuni.2016.02.020 PMC4803910 26982364

[142] Greten FR Arkan MC Bollrath J Hsu LC Goode J Miething C. NF-kappaB is a negative regulator of IL-1beta secretion as revealed by genetic and pharmacological inhibition of IKKbeta. Cell (2007) 130(5):918–31. doi: 10.1016/j.cell.2007.07.009 PMC2134986 17803913

[143] Zhao X Pu D Zhao Z Zhu H Li H Shen Y. Teuvincenone f suppresses LPS-induced inflammation and NLRP3 inflammasome activation by attenuating NEMO ubiquitination. Front Pharmacol (2017) 8:565. doi: 10.3389/fphar.2017.00565 28878677 PMC5572209

[144] Girardin SE Boneca IG Viala J Chamaillard M Labigne A Thomas G. Nod2 is a general sensor of peptidoglycan through muramyl dipeptide (MDP) detection. J Biol Chem (2003) 278(11):8869–72. doi: 10.1074/jbc.C200651200 12527755

[145] Inohara N Ogura Y Fontalba A Gutierrez O Pons F Crespo J. Host recognition of bacterial muramyl dipeptide mediated through NOD2. implications for crohn's disease. J Biol Chem (2003) 278(8):5509–12. doi: 10.1074/jbc.C200673200 12514169

[146] Strober W Watanabe T. NOD2, an intracellular innate immune sensor involved in host defense and crohn's disease. Mucosal Immunol (2011) 4(5):484–95. doi: 10.1038/mi.2011.29 PMC3773501 21750585

[147] Ogura Y Bonen DK Inohara N Nicolae DL Chen FF Ramos R. A frameshift mutation in NOD2 associated with susceptibility to crohn's disease. Nature (2001) 411(6837):603–6. doi: 10.1038/35079114 11385577

[148] Economou M Trikalinos TA Loizou KT Tsianos EV Ioannidis JP. Differential effects of NOD2 variants on crohn's disease risk and phenotype in diverse populations: a metaanalysis. Am J Gastroenterol (2004) 99(12):2393–404. doi: 10.1111/j.1572-0241.2004.40304.x 15571588

[149] Jostins L Ripke S Weersma RK Duerr RH McGovern DP Hui KY. Host-microbe interactions have shaped the genetic architecture of inflammatory bowel disease. Nature (2012) 491(7422):119–24. doi: 10.1038/nature11582 PMC3491803 23128233

[150] Hugot JP Zaccaria I Cavanaugh J Yang H Vermeire S Lappalainen M. Prevalence of CARD15/NOD2 mutations in Caucasian healthy people. Am J Gastroenterol (2007) 102(6):1259–67. doi: 10.1111/j.1572-0241.2007.01149.x 17319929

[151] Li Q Lee CH Peters LA Mastropaolo LA Thoeni C Elkadri A. Variants in TRIM22 that affect NOD2 signaling are associated with very-Early-Onset inflammatory bowel disease. Gastroenterology (2016) 150(5):1196–207. doi: 10.1053/j.gastro.2016.01.031 PMC4842103 26836588

[152] Rosenzweig HL Martin TM Planck SR Galster K Jann MM Davey MP. Activation of NOD2 *in vivo* induces IL-1beta production in the eye *via* caspase-1 but results in ocular inflammation independently of IL-1 signaling. J Leukoc Biol (2008) 84(2):529–36. doi: 10.1189/jlb.0108015 PMC2493069 18495787

[153] Li J Moran T Swanson E Julian C Harris J Bonen DK. Regulation of IL-8 and IL-1beta expression in crohn's disease associated NOD2/CARD15 mutations. Hum Mol Genet (2004) 13(16):1715–25. doi: 10.1093/hmg/ddh182 15198989

[154] Hsu LC Ali SR McGillivray S Tseng PH Mariathasan S Humke EW. A NOD2-NALP1 complex mediates caspase-1-dependent IL-1beta secretion in response to bacillus anthracis infection and muramyl dipeptide. Proc Natl Acad Sci USA (2008) 105(22):7803–8. doi: 10.1073/pnas.0802726105 PMC2409384 18511561

[155] Dahiya Y Pandey RK Sodhi A. Nod2 downregulates TLR2/1 mediated IL1beta gene expression in mouse peritoneal macrophages. PloS One (2011) 6(11):e27828. doi: 10.1371/journal.pone.0027828 22114704 PMC3219683

[156] Amendola A Butera A Sanchez M Strober W Boirivant M. Nod2 deficiency is associated with an increased mucosal immunoregulatory response to commensal microorganisms. Mucosal Immunol (2014) 7(2):391–404. doi: 10.1038/mi.2013.58 23962873 PMC4778708

[157] Umiker B Lee HH Cope J Ajami NJ Laine JP Fregeau C. The NLRP3 inflammasome mediates DSS-induced intestinal inflammation in Nod2 knockout mice. Innate Immun (2019) 25(2):132–43. doi: 10.1177/1753425919826367 PMC6830860 30774010

[158] Rigaud S Fondaneche MC Lambert N Pasquier B Mateo V Soulas P. XIAP deficiency in humans causes an X-linked lymphoproliferative syndrome. Nature (2006) 444(7115):110–4. doi: 10.1038/nature05257 17080092

[159] Speckmann C Lehmberg K Albert MH Damgaard RB Fritsch M Gyrd-Hansen M. X-Linked inhibitor of apoptosis (XIAP) deficiency: the spectrum of presenting manifestations beyond hemophagocytic lymphohistiocytosis. Clin Immunol (2013) 149(1):133–41. doi: 10.1016/j.clim.2013.07.004 23973892

[160] Worthey EA Mayer AN Syverson GD Helbling D Bonacci BB Decker B. Making a definitive diagnosis: successful clinical application of whole exome sequencing in a child with intractable inflammatory bowel disease. Genet Med (2011) 13(3):255–62. doi: 10.1097/GIM.0b013e3182088158 21173700

[161] Zeissig Y Petersen BS Milutinovic S Bosse E Mayr G Peuker K. XIAP variants in male crohn's disease. Gut (2015) 64(1):66–76. doi: 10.1136/gutjnl-2013-306520 24572142

[162] Deveraux QL Takahashi R Salvesen GS Reed JC. X-Linked IAP is a direct inhibitor of cell-death proteases. Nature (1997) 388(6639):300–4. doi: 10.1038/40901 9230442

[163] Duckett CS Nava VE Gedrich RW Clem RJ Van Dongen JL Gilfillan MC. A conserved family of cellular genes related to the baculovirus iap gene and encoding apoptosis inhibitors. EMBO J (1996) 15(11):2685–94. doi: 10.1002/j.1460-2075.1996.tb00629.x PMC450204 8654366

[164] Shiozaki EN Chai J Rigotti DJ Riedl SJ Li P Srinivasula SM. Mechanism of XIAP-mediated inhibition of caspase-9. Mol Cell (2003) 11(2):519–27. doi: 10.1016/S1097-2765(03)00054-6 12620238

[165] Bertrand MJ Doiron K Labbe K Korneluk RG Barker PA Saleh M. Cellular inhibitors of apoptosis cIAP1 and cIAP2 are required for innate immunity signaling by the pattern recognition receptors NOD1 and NOD2. Immunity (2009) 30(6):789–801. doi: 10.1016/j.immuni.2009.04.011 19464198

[166] Damgaard RB Nachbur U Yabal M Wong WW Fiil BK Kastirr M. The ubiquitin ligase XIAP recruits LUBAC for NOD2 signaling in inflammation and innate immunity. Mol Cell (2012) 46(6):746–58. doi: 10.1016/j.molcel.2012.04.014 22607974

[167] Mandal P Berger SB Pillay S Moriwaki K Huang C Guo H. RIP3 induces apoptosis independent of pronecrotic kinase activity. Mol Cell (2014) 56(4):481–95. doi: 10.1016/j.molcel.2014.10.021 PMC4512186 25459880

[168] Tenev T Bianchi K Darding M Broemer M Langlais C Wallberg F. The ripoptosome, a signaling platform that assembles in response to genotoxic stress and loss of IAPs. Mol Cell (2011) 43(3):432–48. doi: 10.1016/j.molcel.2011.06.006 21737329

[169] Marsh RA Madden L Kitchen BJ Mody R McClimon B Jordan MB. XIAP deficiency: a unique primary immunodeficiency best classified as X-linked familial hemophagocytic lymphohistiocytosis and not as X-linked lymphoproliferative disease. Blood (2010) 116(7):1079–82. doi: 10.1182/blood-2010-01-256099 PMC2938130 20489057

[170] Glocker EO Kotlarz D Boztug K Gertz EM Schaffer AA Noyan F. Inflammatory bowel disease and mutations affecting the interleukin-10 receptor. N Engl J Med (2009) 361(21):2033–45. doi: 10.1056/NEJMoa0907206 PMC2787406 19890111

[171] Shouval DS Ouahed J Biswas A Goettel JA Horwitz BH Klein C. Interleukin 10 receptor signaling: master regulator of intestinal mucosal homeostasis in mice and humans. Adv Immunol (2014) 122:177–210. doi: 10.1016/B978-0-12-800267-4.00005-5 24507158 PMC4741283

[172] Bogdan C Vodovotz Y Nathan C. Macrophage deactivation by interleukin 10. J Exp Med (1991) 174(6):1549–55. doi: 10.1084/jem.174.6.1549 PMC2119047 1744584

[173] Ding L Shevach EM. IL-10 inhibits mitogen-induced T cell proliferation by selectively inhibiting macrophage costimulatory function. J Immunol (1992) 148(10):3133–9. doi: 10.4049/jimmunol.148.10.3133 1578140

[174] Murai M Turovskaya O Kim G Madan R Karp CL Cheroutre H. Interleukin 10 acts on regulatory T cells to maintain expression of the transcription factor Foxp3 and suppressive function in mice with colitis. Nat Immunol (2009) 10(11):1178–84. doi: 10.1038/ni.1791 PMC2898179 19783988

[175] Ralph P Nakoinz I Sampson-Johannes A Fong S Lowe D Min HY. IL-10, T lymphocyte inhibitor of human blood cell production of IL-1 and tumor necrosis factor. J Immunol (1992) 148(3):808–14. doi: 10.4049/jimmunol.148.3.808 1730874

[176] Yao Y Vent-Schmidt J McGeough MD Wong M Hoffman HM Steiner TS. Tr1 cells, but not Foxp3+ regulatory T cells, suppress NLRP3 inflammasome activation *via* an IL-10-Dependent mechanism. J Immunol (2015) 195(2):488–97. doi: 10.4049/jimmunol.1403225 26056255

[177] Zhang J Fu S Sun S Li Z Guo B. Inflammasome activation has an important role in the development of spontaneous colitis. Mucosal Immunol (2014) 7(5):1139–50. doi: 10.1038/mi.2014.1 PMC4115056 24472848

[178] Mao L Kitani A Strober W Fuss IJ. The role of NLRP3 and IL-1beta in the pathogenesis of inflammatory bowel disease. Front Immunol (2018) 9:2566. doi: 10.3389/fimmu.2018.02566 30455704 PMC6230716

[179] Aldrich RA Steinberg AG Campbell DC. Pedigree demonstrating a sex-linked recessive condition characterized by draining ears, eczematoid dermatitis and bloody diarrhea. Pediatrics (1954) 13(2):133–9. doi: 10.1542/peds.13.2.133 13133561

[180] Derry JM Ochs HD Francke U. Isolation of a novel gene mutated in wiskott-Aldrich syndrome. Cell (1994) 78(4):635–44. doi: 10.1016/0092-8674(94)90528-2 8069912

[181] Massaad MJ Ramesh N Geha RS. Wiskott-Aldrich syndrome: a comprehensive review. Ann N Y Acad Sci (2013) 1285:26–43. doi: 10.1111/nyas.12049 23527602

[182] Kelly AE Kranitz H Dotsch V Mullins RD. Actin binding to the central domain of WASP/Scar proteins plays a critical role in the activation of the Arp2/3 complex. J Biol Chem (2006) 281(15):10589–97. doi: 10.1074/jbc.M507470200 PMC3077991 16403731

[183] Machesky LM Insall RH. Scar1 and the related wiskott-Aldrich syndrome protein, WASP, regulate the actin cytoskeleton through the Arp2/3 complex. Curr Biol (1998) 8(25):1347–56. doi: 10.1016/S0960-9822(98)00015-3 9889097

[184] Rodnick-Smith M Luan Q Liu SL Nolen BJ. Role and structural mechanism of WASP-triggered conformational changes in branched actin filament nucleation by Arp2/3 complex. Proc Natl Acad Sci USA (2016) 113(27):E3834–43. doi: 10.1073/pnas.1517798113 PMC4941453 27325766

[185] Notarangelo LD Notarangelo LD Ochs HD. WASP and the phenotypic range associated with deficiency. Curr Opin Allergy Clin Immunol (2005) 5(6):485–90. doi: 10.1097/01.all.0000191243.25757.ce 16264326

[186] Folwaczny C Ruelfs C Walther J Konig A Emmerich B. Ulcerative colitis in a patient with wiskott-Aldrich syndrome. Endoscopy (2002) 34(10):840–1. doi: 10.1055/s-2002-34272 12244510

[187] Nguyen DD Maillard MH Cotta-de-Almeida V Mizoguchi E Klein C Fuss I. Lymphocyte-dependent and Th2 cytokine-associated colitis in mice deficient in wiskott-Aldrich syndrome protein. Gastroenterology (2007) 133(4):1188–97. doi: 10.1053/j.gastro.2007.07.010 PMC2048975 17764675

[188] Baehner RL Nathan DG. Leukocyte oxidase: defective activity in chronic granulomatous disease. Science (1967) 155(3764):835–6. doi: 10.1126/science.155.3764.835 6018195

[189] Gungor T Chiesa R. Cellular therapies in chronic granulomatous disease. Front Pediatr (2020) 8:327. doi: 10.3389/fped.2020.00327 32676488 PMC7333593

[190] Heyworth PG Cross AR Curnutte JT. Chronic granulomatous disease. Curr Opin Immunol (2003) 15(5):578–84. doi: 10.1016/S0952-7915(03)00109-2 14499268

[191] Dhillon SS Fattouh R Elkadri A Xu W Murchie R Walters T. Variants in nicotinamide adenine dinucleotide phosphate oxidase complex components determine susceptibility to very early onset inflammatory bowel disease. Gastroenterology (2014) 147(3):680–9.e2. doi: 10.1053/j.gastro.2014.06.005 24931457

[192] Werlin SL Chusid MJ Caya J Oechler HW. Colitis in chronic granulomatous disease. Gastroenterology (1982) 82(2):328–31. doi: 10.1016/0016-5085(82)90023-3 6119271

[193] Cruz CM Rinna A Forman HJ Ventura AL Persechini PM Ojcius DM. ATP activates a reactive oxygen species-dependent oxidative stress response and secretion of proinflammatory cytokines in macrophages. J Biol Chem (2007) 282(5):2871–9. doi: 10.1074/jbc.M608083200 PMC2693903 17132626

[194] Tschopp J Schroder K. NLRP3 inflammasome activation: The convergence of multiple signalling pathways on ROS production? Nat Rev Immunol (2010) 10(3):210–5. doi: 10.1038/nri2725 20168318

[195] Conrad MA Kelsen JR. Genomic and immunologic drivers of very early-onset inflammatory bowel disease. Pediatr Dev Pathol (2019) 22(3):183–93. doi: 10.1177/1093526619834807 PMC6942488 30841788

[196] Taylor H Laurence ADJ Uhlig HH. The role of PTEN in innate and adaptive immunity. Cold Spring Harb Perspect Med (2019) 9(12):a036996. doi: 10.1101/cshperspect.a036996 31501268 PMC6886458

[197] Uhlig HH. Monogenic diseases associated with intestinal inflammation: implications for the understanding of inflammatory bowel disease. Gut (2013) 62(12):1795–805. doi: 10.1136/gutjnl-2012-303956 24203055

[198] Conley ME Mathias D Treadaway J Minegishi Y Rohrer J. Mutations in btk in patients with presumed X-linked agammaglobulinemia. Am J Hum Genet (1998) 62(5):1034–43. doi: 10.1086/301828 PMC1377085 9545398

[199] Guo B Kato RM Garcia-Lloret M Wahl MI Rawlings DJ. Engagement of the human pre-b cell receptor generates a lipid raft-dependent calcium signaling complex. Immunity (2000) 13(2):243–53. doi: 10.1016/S1074-7613(00)00024-8 10981967

[200] Pieper K Grimbacher B Eibel H. B-cell biology and development. J Allergy Clin Immunol (2013) 131(4):959–71. doi: 10.1016/j.jaci.2013.01.046 23465663

[201] Tsukada S Saffran DC Rawlings DJ Parolini O Allen RC Klisak I. Deficient expression of a b cell cytoplasmic tyrosine kinase in human X-linked agammaglobulinemia. Cell (1993) 72(2):279–90. doi: 10.1016/0092-8674(93)90667-F 8425221

[202] Vetrie D Vorechovsky I Sideras P Holland J Davies A Flinter F. The gene involved in X-linked agammaglobulinaemia is a member of the src family of protein-tyrosine kinases. Nature (1993) 361(6409):226–33. doi: 10.1038/361226a0 8380905

[203] Weber ANR. Targeting the NLRP3 inflammasome *via* BTK. Front Cell Dev Biol (2021) 9:630479. doi: 10.3389/fcell.2021.630479 33718366 PMC7947255

[204] Dupuis S Dargemont C Fieschi C Thomassin N Rosenzweig S Harris J. Impairment of mycobacterial but not viral immunity by a germline human STAT1 mutation. Science (2001) 293(5528):300–3. doi: 10.1126/science.1061154 11452125

[205] Mogensen TH. IRF. And STAT transcription factors - from basic biology to roles in infection, protective immunity, and primary immunodeficiencies. Front Immunol (2018) 9:3047. doi: 10.3389/fimmu.2018.03047 30671054 PMC6331453

[206] Uzel G Sampaio EP Lawrence MG Hsu AP Hackett M Dorsey MJ. Dominant gain-of-function STAT1 mutations in FOXP3 wild-type immune dysregulation-polyendocrinopathy-enteropathy-X-linked-like syndrome. J Allergy Clin Immunol (2013) 131(6):1611–23. doi: 10.1016/j.jaci.2012.11.054 PMC3672257 23534974

[207] Guarda G Braun M Staehli F Tardivel A Mattmann C Forster I. Type I interferon inhibits interleukin-1 production and inflammasome activation. Immunity (2011) 34(2):213–23. doi: 10.1016/j.immuni.2011.02.006 21349431

[208] Rauch I Muller M Decker T. The regulation of inflammation by interferons and their STATs. JAKSTAT (2013) 2(1):e23820. doi: 10.4161/jkst.23820 24058799 PMC3670275

[209] Blaydon DC Biancheri P Di WL Plagnol V Cabral RM Brooke MA. Inflammatory skin and bowel disease linked to ADAM17 deletion. N Engl J Med (2011) 365(16):1502–8. doi: 10.1056/NEJMoa1100721 22010916

[210] Adu-Amankwaah J Adzika GK Adekunle AO Ndzie Noah ML Mprah R Bushi A. ADAM17, a key player of cardiac inflammation and fibrosis in heart failure development during chronic catecholamine stress. Front Cell Dev Biol (2021) 9:732952. doi: 10.3389/fcell.2021.732952 34966735 PMC8710811

[211] Black RA Rauch CT Kozlosky CJ Peschon JJ Slack JL Wolfson MF. A metalloproteinase disintegrin that releases tumour-necrosis factor-alpha from cells. Nature (1997) 385(6618):729–33. doi: 10.1038/385729a0 9034190

[212] Moss ML Jin SL Milla ME Bickett DM Burkhart W Carter HL. Cloning of a disintegrin metalloproteinase that processes precursor tumour-necrosis factor-alpha. Nature (1997) 385(6618):733–6. doi: 10.1038/385733a0 9034191

[213] McGeough MD Wree A Inzaugarat ME Haimovich A Johnson CD Pena CA. TNF regulates transcription of NLRP3 inflammasome components and inflammatory molecules in cryopyrinopathies. J Clin Invest (2017) 127(12):4488–97. doi: 10.1172/JCI90699 PMC5707143 29130929

[214] Sharma D Malik A Guy C Vogel P Kanneganti TD. TNF/TNFR axis promotes pyrin inflammasome activation and distinctly modulates pyrin inflammasomopathy. J Clin Invest (2019) 129(1):150–62. doi: 10.1172/JCI121372 PMC6307946 30457980

